# The microbial communities (bacteria, algae, zooplankton, and fungi) improved biofloc technology including the nitrogen-related material cycle in *Litopenaeus vannamei* farms

**DOI:** 10.3389/fbioe.2022.883522

**Published:** 2022-11-23

**Authors:** Hyun-Sik Yun, Dong-Hyun Kim, Jong-Guk Kim, Young-Saeng Kim, Ho-Sung Yoon

**Affiliations:** ^1^ Department of Biology, College of Natural Sciences, Kyungpook National University, Daegu, South Korea; ^2^ School of Applied Biosciences, Kyungpook National University, Daegu, South Korea; ^3^ School of Life Sciences and Biotechnology, BK21 Plus KNU Creative BioResearch Group, Kyungpook National University, Daegu, South Korea; ^4^ Research Institute of Ulleung-do & Dok-do, Kyungpook National University, Daegu, South Korea; ^5^ Advanced Bio-Resource Research Center, Kyungpook National University, Daegu, South Korea

**Keywords:** biofloc technology, illumina MiSeq, microbial community, shrimp farm, zoo plankton

## Abstract

Microbes are essential in biofloc technology for controlling nitrogen levels in water. The composition and function of microorganisms with biofloc systems were reported; however, data on microorganisms other than bacteria, such as algae (which are essential in the nitrogen cycle) and zooplankton (which are bacterial and algal predators), remain limited. The microbial communities (including bacteria, algae, zooplankton, and fungi) were investigated in shrimp farms using biofloc technology. Using Illumina MiSeq sequencing, the V4 region of 18S rRNA and the V3–V4 region of 16S rRNA were utilized for the analysis of the eukaryotic and prokaryotic microbial communities. As a result, it was found that the biofloc in the shrimp farm consisted of 48.73%–73.04% eukaryotic organisms and 26.96%–51.27% prokaryotic organisms. In these shrimp farms, prokaryotic microbial communities had higher specie richness and diversity than eukaryotic microbial communities. However, the eukaryotic microbial communities were more abundant than their prokaryotic counterparts, while algae and zooplankton dominated them. It was discovered that the structures of the microbial communities in the shrimp farms seemed to depend on the effects of predation by zooplankton and other related organisms. The results provided the nitrogen cycle in biofloc systems by the algal and bacterial groups in microbial communities.

## 1 Introduction

Given their roles as decomposers and producers, microorganisms play crucial roles in various ecosystem material cycles (Mickalide and Kuehn, 2019; López-Mondéjar et al., 2020). This includes the nitrogen-related material cycle (Cirri and Pohnert, 2019; Urakawa et al., 2019), in which some microorganisms utilize nitrogen for anabolic or catabolic processes (Takai, 2019; Dai et al., 2020). Through such methods, pollutants, such as ammonia, are removed, and nitrogen-based compounds, e.g., proteins, are synthesized (Pilgrim et al., 1970). The action of microorganisms in this context has become the basis for their use on an industrial level (Huo et al., 2020; Zhang et al., 2020). For example, microbial functions are applied to treat nitrogen compounds in wastewater generated in cities (Huo et al., 2020; Zhang et al., 2020). Additionally, microorganisms are used in removing nitrogen-based compounds from water containing aquatic organisms in large-scale fish farms to small-scale home aquariums (Miranda-Baeza et al., 2020; Putra et al., 2020). However, until recently, microorganism-based removal of pollutants was rarely used in aquatic organism-breeding programs; indeed, the breeding of aquatic organisms was traditionally sustained through water exchange (Timmons et al., 1998). Given that the water exchange method needs substantial water levels and generates wastewater, improved methods are now being developed (Timmons et al., 1998).

Biofloc technology is a method that removes some limitations associated with traditional aquatic organism-breeding methods (Bakar et al., 2015; Chen et al., 2019). With biofloc technology, pollutants, including ammonia generated by aquatic organisms, are removed from water by culturing microorganisms in the aquatic-organisms’ breeding space (Bakar et al., 2015; Chen et al., 2019). These cultured microorganisms remove pollutants and act as food for aquatic organisms (Cardona et al., 2015; Bossier and Ekasari, 2017). Consequently, the microbial-derived biomass reduces the necessary feed input during the breeding of aquatic organisms and increases productivity (Cardona et al., 2015; Bossier and Ekasari, 2017). Moreover, effective removal of pollutants using microorganisms has made the traditional water exchange process unnecessary (Bakar et al., 2015; Bossier and Ekasari, 2017; Chen et al., 2019). Therefore, biofloc technology has reduced costs and increased efficiency in the aquatic organism production and breeding industry (Gaitán-Angulo et al., 2016).

To maximize the effects of biofloc technology, previous research on the constituents of microbial communities in biofloc systems has been conducted (Wei G et al., 2020; Wei Y-F et al., 2020). Through previous studies, it has been found that the structural characteristics of the microbial community constituting biofloc can vary depending on the method and carbon source applied to form biofloc (Vilani et al., 2016; Arantes et al., 2017; Luo et al., 2022). Furthermore, this revealed the need for a deeper understanding of the characteristics of photo-autotrophic, heterotrophic, and chemotrophic microbial organisms in biofloc (Vilani et al., 2016; Arantes et al., 2017; Luo et al., 2022). Although previous pollutant removal and floc formation-related research has enhanced understanding of biofloc technology (D’Silva and Kyndt, 2020; Dauda, 2020), more studies on microalgae are needed to deepen this understanding (Ray et al., 2009; Wang et al., 2019). It is known that microalgae can synthesize nitrogen-related organic substances, such as proteins, using ammonia and nitrate (Cui et al., 2020). Additionally, hydrocarbons and lipids (including unsaturated fatty acids) can be produced through photosynthesis, while antioxidants such as astaxanthin and lutein are included among the pigments manufactured for photosynthesis (Kawale and Kishore, 2019; Cui et al., 2020; Li L. et al., 2020). These microalgae-produced substances can be fed to aquatic organisms, thereby increasing the productivity and quality of farm products (Crab et al., 2012; Dauda, 2020; Khanjani and Sharifinia, 2020). However, more research on the role and features of microalgae in biofloc technology is still needed.

In the Republic of Korea, biofloc technology is applied to various aquatic organisms, including the shrimp species *Litopenaeus vannamei*, which is now actively manufactured with the use of biofloc (Taw, 2014; Hamidoghli et al., 2018). In this study, Illumina MiSeq system was applied to characterize the microbial communities that constitute the biofloc in shrimp farms of the southern Korean Peninsula. The structure of these communities, including eukaryotic and prokaryotic microbial communities, was analyzed. The algal and bacterial groups demonstrate the metabolic processes involved in the nitrogen cycle in biofloc systems.

## 2 Materials and methods

### 2.1 Sample collection

**FIGURE 1:**
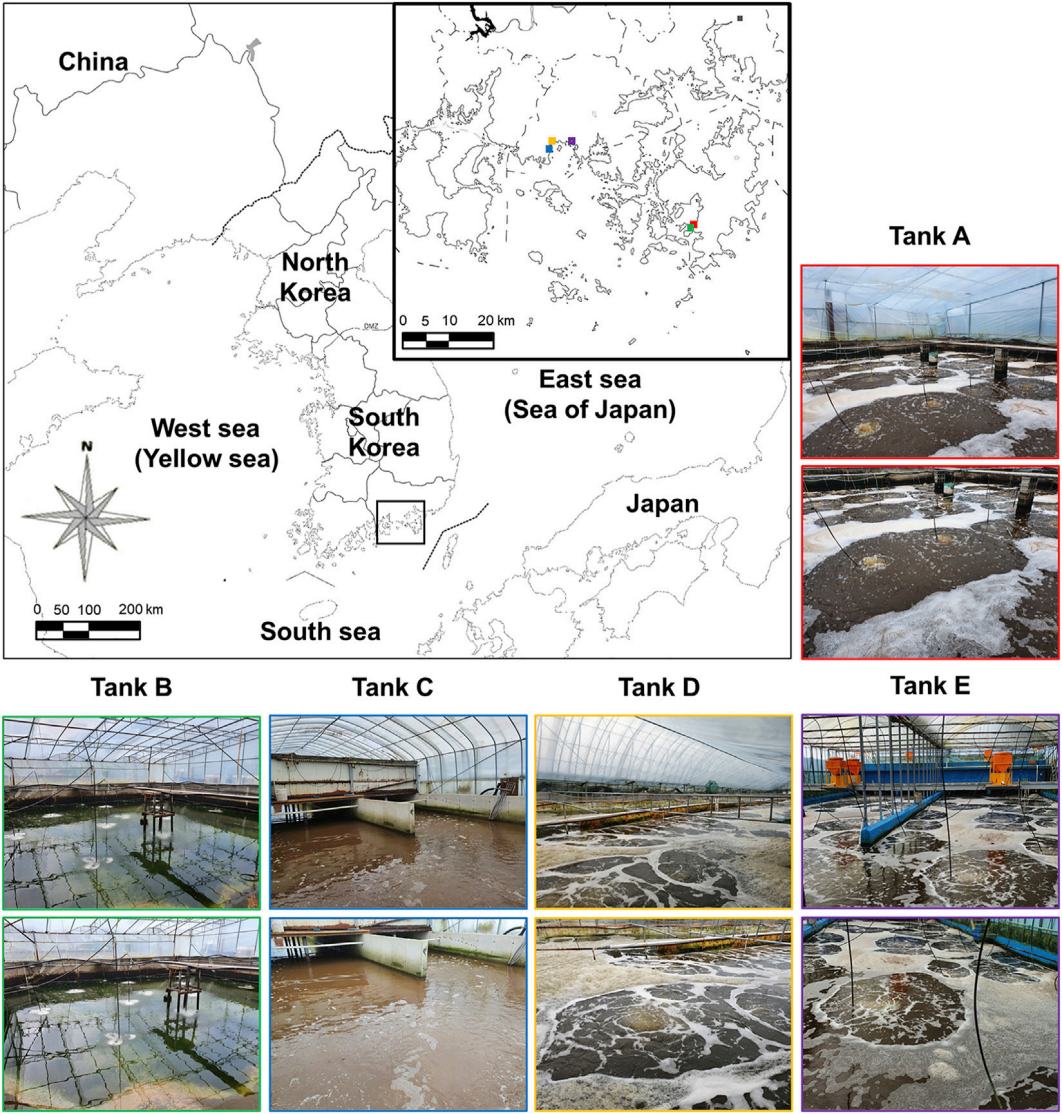
Sampling sites on the southern coast of the Korean Peninsula. The location of the *Litopenaeus vannamei* shrimp farms using biofloc technology is indicated by the black box. Additionally, locations (colored squares on the main map) and images of tanks A (red boxes), B (green boxes), C (blue boxes), D (orange boxes), and E (purple boxes) are indicated. For exact tank locations, see Section 2.1.

Water samples from the shrimp farms were obtained from five farm tanks (Figure 1; Tank A: 34°47′33.1″N 128°34′17.5″E, 2456, Geojenamseo-ro, Dongbu-myeon, Geoje-si, Gyeongsangnam-do, Republic of Korea; Tank B: 34°47′33.2″N 128°34′17.8″E, 2456, Geojenamseo-ro, Dongbu-myeon, Geoje-si, Gyeongsangnam-do, Republic of Korea; Tank C: 34°56′08.7″N 128°11′44.8″E, 1656, Jaranman-ro, Hail-myeon, Goseong-gun, Gyeongsangnam-do, Republic of Korea; Tank D: 34°56′19.7″N 128°11′50.5″E, 1699, Jaranman-ro, Hail-myeon, Goseong-gun, Gyeongsangnam-do, Republic of Korea; Tank E: 34°56′36.1″N 128°14′53.1″E, 199, Sambong 1-gil, Samsan-myeon, Goseong-gun, Gyeongsangnam-do, Republic of Korea). The tanks of each farm are designed and modified according to previous research (Avnimelech, 1999; Arnold et al., 2009; Khanjani and Sharifinia, 2020). The pumps and air stones were used to circulate the water inside the tank, and molasses (48% total carbon, EMzone, Thailand) were added to the tank water for the formation of a biofloc-forming microbial community and proceeded according to the calculation guided (Avnimelech, 1999; Arnold et al., 2009; Khanjani and Sharifinia, 2020). The farm maintains a shrimp density of 250–350 individuals/m^2^. The time when biofloc was investigated was nearing the harvest time of shrimp. Shrimp purchased from shrimp farms and weighed for each shrimp was found to be in the range of 25–30 g for each shrimp from all shrimp farms. To form and maintain bioflocs, all of the shrimp farms investigated were using molasses. The water in the tanks was sampled and the floc derived from the microorganisms contained in the water. Five water samples were taken from five sites in each tank. All samples (500-ml water) were collected on 15 June 2020, and stored in refrigerated containers before being transported to the laboratory. From these main samples, further samples were taken on 16 June 2020, for Illumina MiSeq analysis, which was conducted by Macrogen Co., Ltd. (Seoul, South Korea; https://dna.macrogen.com/kor/), with samples delivered through an express courier service.

### 2.2 Measurement of physicochemical environmental factors

A multiparameter instrument (U-50 Multiparameter Water Quality Meter; HORIBA, Kyoto, Japan) was used to determine the physicochemical and environmental factors of the shrimp farm-water samples. Samples for measuring physicochemical and environmental factors were collected on the same day and at the same time separately from samples for Illumina MiSeq analysis. The measurement was carried out immediately after sampling, 500-ml samples were placed in measuring tubes and evaluated for seven measurement factors: temperature, pH, electrical conductivity (EC), dissolved oxygen (DO), nephelometric turbidity unit (NTU), total dissolved solids (TDS), and salinity.

### 2.3 Illumina MiSeq for microbial community analysis

Illumina MiSeq analysis was conducted in the laboratory of Macrogen (Vo and Jedlicka 2014). First, DNA was extracted using a DNeasy PowerSoil Kit (Cat. No. 12888-100, QIAGEN) according to the manufacturer’s instructions (Claassen et al., 2013). Next, the quality of the extracted DNA was quantified using PicoGreen (Promega) and a Nanodrop system. PCR amplified each DNA sample, and the amplified samples were prepared according to either the Illumina 18S Metagenomic Sequencing Library protocol or the Illumina 16S Metagenomic Sequencing Library protocol for eukaryotic or prokaryotic microbial communities, respectively (Stoeck et al., 2010; Klindworth et al., 2013; Vo and Jedlicka, 2014). The 18S rRNA region was amplified using the 18S V4 primer set (forward primer: TAReuk454FWD1, 5′-CCAGCA (G⁄C)C(C⁄T)GCGGTAA TTCC-3’; reverse primer: TAReukREV3, 5′-ACTTTCG TTCTTGAT (C⁄T) (A⁄G)A-3′) (Stoeck et al., 2010); the 16S rRNA region was amplified using the 16S V3–V4 primer set (forward primer: MiSeq341F, 5′-TCGTCGGCAGCGTC AGATGTGTATAAGAGA CAGCCTACGGGNGGCWGCAG-3’; reverse primer: MiSeq805R, 5′-GTCTCGT GGGCTCGGAGATGTGTATAAGAGACAGGACTACHVGGGTATCTAATCC-3′) (Klindworth et al., 2013). The quality of the amplified DNA was quantified using PicoGreen and a VICTOR Nivo system (PerkinElmer). Subsequent limited-cycle amplification was performed to add multiplexing indices and Illumina sequencing adapters to the amplified PCR products (Meyer and Kircher, 2010). The final products were normalized and pooled using PicoGreen, and the size of the libraries was verified using TapeStation DNA D1000 ScreenTape system (Agilent, Santa Clara, CA, United States). Subsequently, the sequencing data were analyzed using the MiSeq platform (Illumina, San Diego, CA, United States) (Kozich et al., 2013).

### 2.4 Taxonomic identification

The raw data from MiSeq were demultiplexed using the index sequence, and a FASTQ file was generated for each sample. The adapter sequence was eliminated using SeqPurge, and the barcode sequence was trimmed and filtered according to the standard quality value (low-quality sequences: average quality value <25) (Sturm et al., 2016). Based on the barcode sequences included in the NCBI database, all refined raw data were identified using a BLASTN search (query coverage: > 99%) (Zhang Z et al., 2000). When the results could not be classified into a sublevel, ‘-’ was added to the end of the name. The operational taxonomic unit (OTU) was determined by CD-HIT at a 97% sequence similarity level (Li et al., 2012). Rarefaction curves and diversity indicators were computed through the mothur platform (Heck et al., 1975; Schloss et al., 2009). The results of beta diversity (sample diversity information of the comparison group) based on weighted UniFrac distance flexibility between the samples were visualized using an UPGMA tree (FigTree, http://tree.bio.ed.ac.uk/software/figtree/) (Bokulich et al., 2013).

### 2.5 Statistical analysis

We expressed the ratio of biomass present and the fatty acids contents, which we defined as 100%. We compared individual data points using Student’s *t*-test, and a *p*-value of <0.05 was considered statistically significant. All experiments were performed at least in triplicate, and the general microbiology test data were expressed as mean ± standard deviation (SD) (n = 3).

## 3 Results and Discussion

### 3.1 Physicochemical Characteristics of the shrimp farm aquatic environment

The physicochemical and environmental features of the aquatic environment in the shrimp farms are summarized in Table 1. Temperatures in tanks were 24.40°C–28.20°C (highest: tank A, 28.20°C; lowest: tank D, 24.40°C; range: 3.80°C). For pH, there was a difference of 1.76 between the highest (tank B, 8.51) and lowest (tank D, 6.75) pH values. In all tanks, EC was around 50.0 mS/cm (highest: tank E, 51.3 mS/cm; lowest: tank A, 49.6 mS/cm; range: 1.7 mS/cm). DO in tanks was 7.05–7.86 mg/L (highest: tank D, 7.86 mg/L; lowest: tank E, 7.05 mg/L; range: 0.81 mg/L). The NTU values in samples were 4.25–95.10 NTU (highest: tank C, 95.10 NTU; lowest: tank B, 4.25 NTU; range: 90.85 NTU); thus, of all measured features, NTU varied most among tanks. All measured TDS values were around 30.0-g/L (highest: tank E, 30.8 g/L; lowest: tank A and tank D, 30.3-g/L; range: 0.5 g/L). Salinity was around 33.0‰ in all tanks (highest: tank E, 33.7‰; lowest: tank A, 32.5‰; range: 1.2‰). From these features, the differences among tanks were insignificant for four measured factors, namely EC, DO, TDS, and salinity. Still, they were significant for three measured factors, i.e., temperature, pH, and NTU.

**TABLE 1:** The seven physicochemical factors studied in the five tanks from *Litopenaeus vannamei* shrimp farms.

	Tank A	Tank B	Tank C	Tank D	Tank E
Temperature (°C)	28.20	24.80	25.47	24.40	26.42
pH	7.04	8.51	6.83	6.75	7.83
EC (mS/cm)	49.6	50.8	49.9	50.5	51.3
DO (mg/L)	7.21	7.62	7.66	7.86	7.05
Turbidity (NTU)	84.80	4.25	95.10	86.30	32.80
TDS (g/L)	30.3	30.5	30.4	30.3	30.8
Salinity (%)	32.5	33.3	32.7	33.1	33.7

EC: electrical conductivity; DO: dissolved oxygen; NTU: nephelometric turbidity unit; TDS: total dissolved solids.

In this study, the five tanks exhibited similar results for all environmental features except pH and turbidity. Of all environmental features with similar results, the error range was <10%. In contrast, the error range for pH and turbidity was >10%. Additionally, the tendency of measuring low turbidity in samples with high pH was confirmed. Generally, pH can substantially affect aquatic organisms, but it can also be altered by several microorganisms, including bacteria and algae (Shiraiwa et al., 1993; García-de-la-Fuente et al., 2011; Giordani et al., 2019). The observed differences in pH propose that the composition of microbial communities differed among samples and reflects the compositional features of the community (Lei and VanderGheynst, 2000; Giordani et al., 2019). The features of the microbial community are also indicated by turbidity, which arises in phenomena such as flocculation (Van Den Hende et al., 2011). In summary, the high pH and low turbidity in this study suggest that the microbial communities of shrimp farms using biofloc were affected by pH, which altered turbidity.

### 3.2 Environmental characteristics and microbial species diversity in shrimp farms using biofloc systems

**FIGURE 2:**
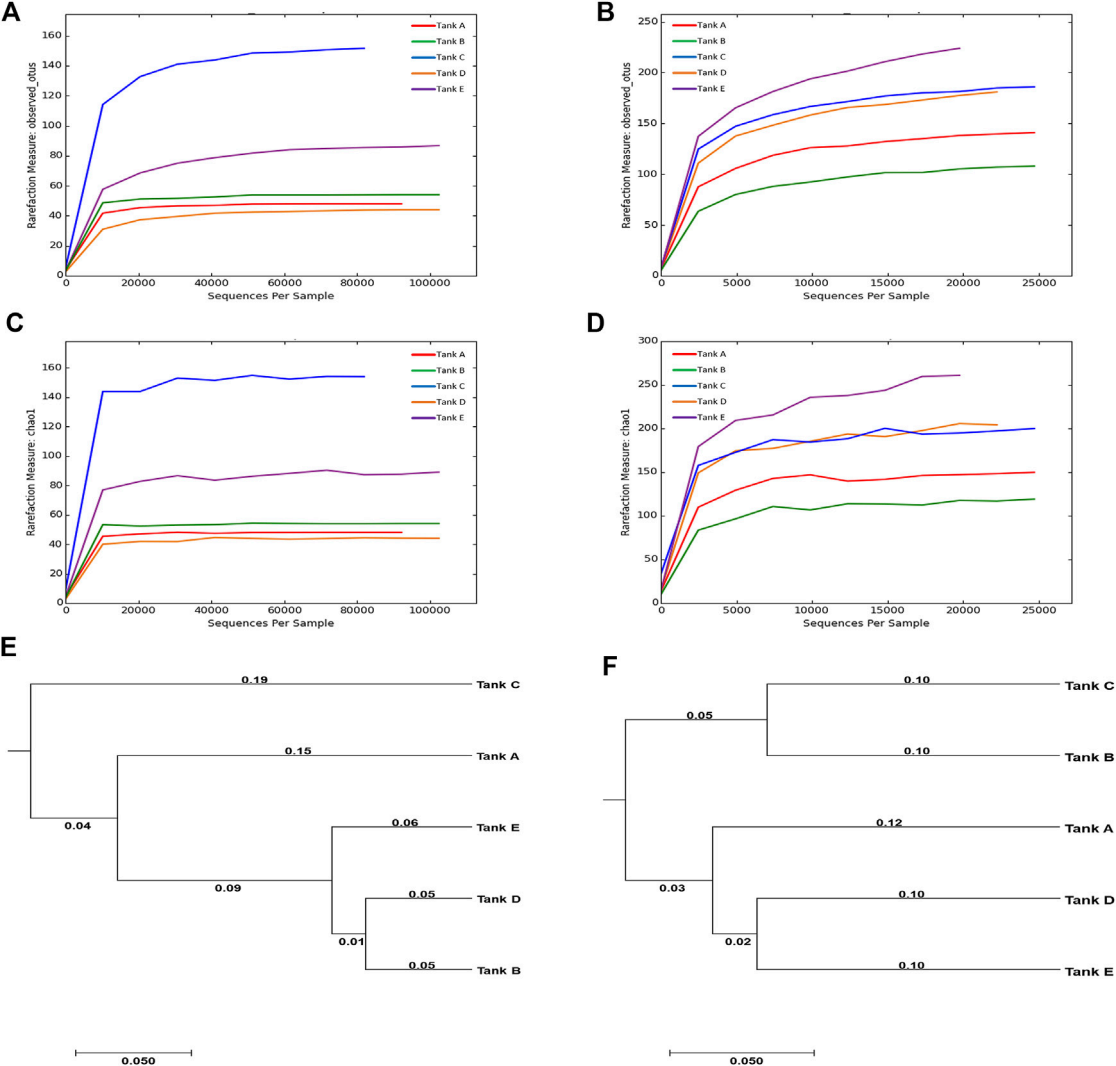
Rarefaction curves and UPGMA trees calculated from the five tanks. **(A–D)** Rarefaction curves are for OTUs and species richness (Chao1). The OTUs were analyzed using CD-HIT program at 97% sequence similarity, and the rarefaction curves and diversity indices for OTUs and Chao1 were computed using the mothur platform. **(E,F)** The relationships among the microbial community diversities for the five tanks. The UPGMA trees were formed using the weighted UniFrac distance. [For eukaryotic microbial communities: rarefaction curves of **(A)** OTUs and **(C)** Chao1, and **(E)** UPGMA tree. For prokaryotic microbial communities: rarefaction curves of **(B)** OTUs and **(D)** Chao1, and **(F)** UPGMA tree]. The data underlying all the graphs indicated in this figure can be found in the Supplementary Table S3.

The results of Illumina MiSeq, and the species richness and diversity of the microbial community (calculated based on sequencing results), are indicated in Supplementary Table S3 and Figure 2. In the analyzed samples, 90,416–120,981 total reads were obtained for the eukaryotic microbial community depending on the tank, of which 23,414–70,926 reads were validated. Although tank E had the largest number of total reads (120,981) among all tanks, the difference between the total and validated, reads was also the largest (difference: 96,575). In contrast, tank B had the least number of total reads (90,416) with the least difference between total and validated reads (difference: 19,490). For the prokaryotic microbial community, 22,060–41,788 total reads were obtained from samples depending on the tank, of which 21,321–41,533 reads were validated. The total and validated reads were highest in tank B (41,788 and 41,533, respectively) and lowest in tank E (22,060 and 21,321, respectively). Unlike in the eukaryotic microbial community, the difference between the total and validated reads was relatively small in the prokaryotic microbial community (differences: tank A, 72; tank B, 255; tank C, 1,505; tank D, 955; tank E, 739). The mean read-length of the analyzed reads was 402.90–416.43 bp for the eukaryotic microbial community and 438.33–450.89 bp for the prokaryotic microbial community. The maximum read-lengths for the eukaryotic and prokaryotic microbial communities were 419 and 461 bp, respectively. The number of OTUs detected from the samples was 44–152 for the eukaryotic microbial community and 115–228 for the prokaryotic microbial community. Thus, across all samples, the number of OTUs was greater in the prokaryotic microbial community than in the eukaryotic microbial community.

In the microbial communities of the shrimp farms using biofloc technology, the scale of the eukaryotic microbial community was relatively larger than that of the prokaryotic microbial community; however, there was more species richness and diversity in the prokaryotic microbial community. Comparing the eukaryotic or prokaryotic microbial communities in each sample, no specific patterns among the number of reads, species richness, and species diversity was found. These results support previous findings of lack of association between community scale, species richness, and species diversity (Sanjit and Bhatt, 2005). Additionally, no obvious relationship between the eukaryotic microbial community and prokaryotic microbial community were found in samples (Santi et al., 2019), e.g., the number of eukaryotic microbial OTUs was highest in tank C (152), but the number of prokaryotic microbial OTUs was highest in tank E (228); the number of eukaryotic microbial OTUs was lowest in tank D (44), but the number of prokaryotic microbial OTUs in tank D was relatively high (183).

Chao1, an indicator of species richness, exhibited a similar trend to that observed in OTUs. In the eukaryotic microbial community, species richness was lowest in tank D (44.00) and highest in tank C (154.50). In the prokaryotic microbial community, species richness was generally higher; it was lowest in tank B (137.75) and highest in tank E (276.24). When considering the same sample, the species richness of the eukaryotic microbial community was lower than that of the prokaryotic microbial community. Two diversity indicators (Shannon and inverse Simpson) were used to quantify species diversity; however, results for the two diversity indicators were inconsistent. In the eukaryotic microbial community, the Shannon value was ordered as follows: tank C (2.83) > tank A (2.37) > tank B (2.00) > tank E (1.55) > tank D (1.36). In contrast, the inverse Simpson value was ordered as follows: tank A (0.73) > tank C (0.68) > tank B (0.57) > tank D (0.53) > tank E (0.41). Among the analyzed samples, the eukaryotic microbial communities of tank A (Shannon, 2.37; inverse Simpson, 0.73) and tank C (Shannon, 2.83; inverse Simpson, 0.68) exhibited relatively high species diversity, while the species diversity of tank D (Shannon, 1.36; inverse Simpson, 0.53) and tank E (Shannon, 1.55; inverse Simpson, 0.41) was relatively low. In the prokaryotic microbial community, Shannon values were ordered as follows: tank C (5.03) > tank E (4.56) > tank D (4.52) > tank A (4.35) > tank B (3.11). In contrast, inverse Simpson values in this community were as follows: tank C (0.92) > tank D (0.91) > tank A (0.90) > tank E (0.88) > tank B (0.68). In the prokaryotic microbial community, tank C had relatively high species diversity (Shannon, 5.03; inverse Simpson, 0.92), while the species diversity of tank B (Shannon, 3.11; inverse Simpson, 0.68) was low.

Based on UPGMA trees, the eukaryotic microbial community of tank B was similar only to that of tank D. In contrast, the prokaryotic microbial community of tank B was comparable only to that of tank C. The fact that the microbial community of tank B exhibited no similarities with other tank communities seemed to be due to differences in tank B’s pH and turbidity relative to other tanks (Santi et al., 2019). Based on these results, it was suggest that differences in microbial communities can occur when environmental features, such as pH and turbidity, differ (Santi et al., 2019). The similarity between microbial communities was visualized using an UPGMA tree (Figures 2E,F). Throughout the eukaryotic and prokaryotic microbial communities, similarities were high in the order tank E, tank A, and tank C based on tank D. For tank B, the eukaryotic microbial community was highly similar to that of tank D. In contrast, the prokaryotic microbial community was highly similar to that of tank C. These results indicate that there were no association between the number of reads, species richness, and diversity in the microbial communities from the biofloc of the shrimp farms. Additionally, species richness and diversity had little relevance to the similarity between the microbial communities. Even though the number of reads analyzed from the prokaryotic microbial community was less than that from the eukaryotic microbial community, the species richness and diversity were higher.

In the results, unique microbial communities were formed in each shrimp farm using biofloc according to the subtle environmental differences related to each sample. Although the features of the unique microbial community did not manifest as differences in species richness and diversity, they are expected to be revealed by the similarities between microbial communities. Additionally, prokaryotic microbial communities tend to have higher species richness and diversity than eukaryotic microbial communities, but one community type did not influence the diversity of the other.

### 3.3 Compositional characteristics of the microbial communities in shrimp farms with biofloc technology

Microbial organisms in shrimp farms using biofloc systems can decompose nitrogen compounds, including ammonia, or convert them into substances that are nontoxic to aquatic organisms (Abakari et al., 2020). This function is conducted by bacteria involved in some denitrification and nitrification processes or by an algal group that synthesizes organic compounds containing nitrogen through photosynthesis (Ray et al., 2009; Abakari et al., 2020; Luo et al., 2020). According to the results, there are relatively large-scale eukaryotic microbial communities with abundant algal or zooplankton/other groups in the studied shrimp farms using biofloc technology. Therefore, the biological mechanisms related to nitrogen compounds are expected to have different properties depending on the composition of the microbial community (Ray et al., 2009; Abakari et al., 2020). In microbial communities, in which the eukaryotic microbial community is dominated by zooplankton/other related organisms (such as in tanks A, C, and E) the mechanisms related to nitrogen compounds would be dependent on the bacterial group (Ray et al., 2009; Abakari et al., 2020). Alternatively, where the algal group occupies most of the eukaryotic microbial community (such as in tanks B and D, in which algae also occupied more than 50% of the total microbial community), the metabolic process would be dominated by the algal group (Ray et al., 2009).

**FIGURE 3:**
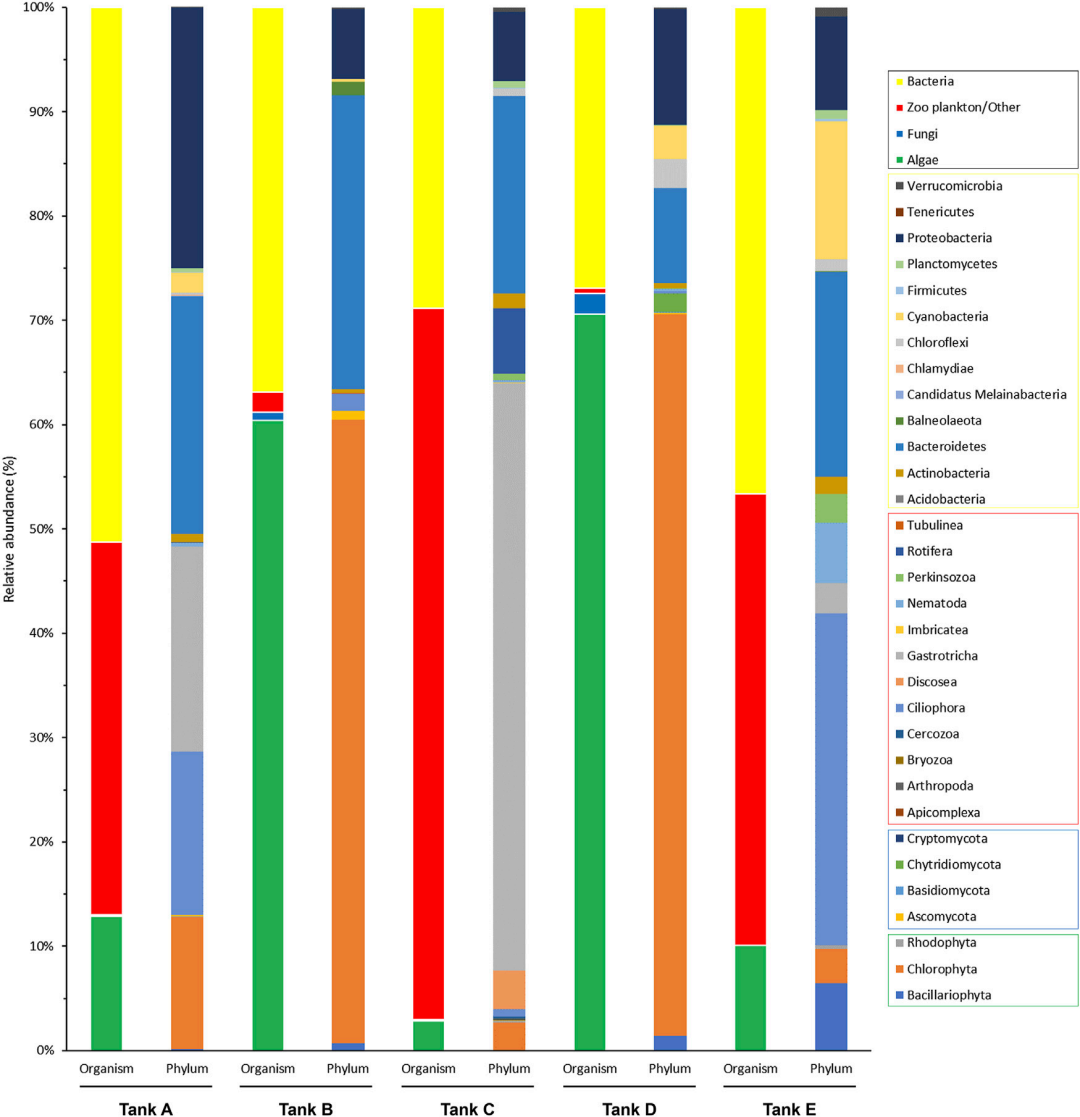
Community composition of the organism groups and phyla from the five tanks. The four organism groups that composed the microbial communities in each tank and the phyla from each group are indicated (black box, organism groups; yellow box, bacterial phyla; red box, zooplankton/other related phyla; blue box, fungal phyla; green box, algal phyla). The data underlying all the graphs indicated in this figure can be found in Supplementary Tables S1, S2.

Based on the results of taxonomic identification, members of eukaryotic and prokaryotic microbial communities was classified into four organism groups (algae, fungi, zooplankton/other, and bacteria), and the phylum composition of each group were compared (Figure 3). In tank A, the microbial community comprised 12.89% algae, 0.08% fungi, 35.76% zooplankton/other groups, and 51.27% bacteria. In tank B, it consisted of 60.38% algae, 0.82% of fungi, 1.95% of zooplankton/other groups, and 36.85% bacteria. In tank C, it comprised 2.81% algae, 0.16% fungi, 68.21% zooplankton/other groups, and 28.82% bacteria. In tank D, it consisted of 70.60% algae, 1.96% fungi, 0.48% zooplankton/other groups, and 26.96% bacteria. Finally, in tank E, it comprised 10.49% algae, 0.02% fungi, 42.86% zooplankton/other groups, and 46.62% bacteria. Based on these findings, the microbial community was composed of 48.73%–73.04% eukaryotic organisms (i.e., algae, fungi, and zooplankton/other groups) and 26.96%–51.27% prokaryotic organisms (bacteria). Therefore, there was a more abundant eukaryotic microbial community in the analyzed samples. Furthermore, the features of the eukaryotic microbial community were divided into two types: algal group-dominant (tanks B and D) and zooplankton/other group-dominant (tanks A, C, and E). Notably, fungi were detected at low levels in all samples (0.02%–1.96%).

**FIGURE 4:**
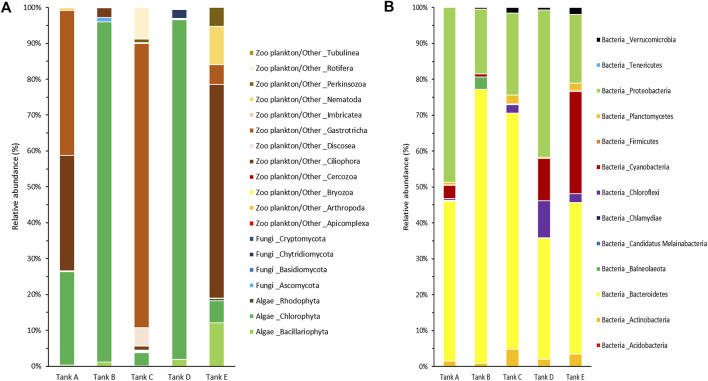
Community composition at the phylum level from the five tanks. **(A)** The eukaryotic and **(B)** prokaryotic microbial communities are indicated separately. The data underlying all the graphs indicated in this figure can be found in Table 2 and Supplementary Tables S1, S2.

For a more detailed among-sample comparison of the eukaryotic and prokaryotic microbial communities, their compositions at the phylum level were analyzed separately (Figure 4). In total, 19 phyla were detected in the eukaryotic microbial community (five samples); they consisted of three algal phyla (*Bacillariophyta, Chlorophyta, and Rhodophyta*), four fungal phyla (*Ascomycota, Basidiomycota, Chytridiomycota, and Cryptomycota*), and 12 zooplankton/other related phyla (*Apicomplexa, Arthropoda, Bryozoa, Cercozoa, Ciliophora*, *Discosea*, *Gastrotricha*, *Imbricatea, Nematoda, Perkinsozoa, Rotifera*, and *Tubulinea*) (Figure 4A). In tank A, three algal phyla (*Bacillariophyta*, 0.31%; *Chlorophyta*, 25.99%; *Rhodophyta*, 0.16%), one fungal phylum (*Ascomycota*, 0.17%), and four zooplankton/other related phyla (*Ciliophora*, 32.13%; *Gastrotricha*, 40.44%; *Nematoda*, 0.66%; *Rotifera*, 0.14%) were detected. In tank B, two algal phyla (*Bacillariophyta*, 1.16%; *Chlorophyta*, 94.78%), two fungal phyla (*Ascomycota*, 1.29%; *Basidiomycota*, 0.01%), and two zooplankton/other related phyla (*Ciliophora*, 2.63%; *Tubulinea*, 0.13%) were detected. In tank C, three algal phyla (*Bacillariophyta*, 0.07%; *Chlorophyta*, 3.72%; *Rhodophyta*, 0.16%), three fungal phyla (*Ascomycota*, 0.14%; *Basidiomycota*, < 0.01%; *Cryptomycota*, 0.09%), and 11 zooplankton/other related phyla (*Apicomplexa*, 0.03%; *Arthropoda*, 0.02%; *Bryozoa*, 0.01%; *Cercozoa*, 0.32%; *Ciliophora*, 0.99%; *Discosea*, 5.22%; *Gastrotricha*, 79.14%; *Imbricatea*, 0.02%; *Nematoda*, 0.31%; *Perkinsozoa*, 0.96%; *Rotifera*, 8.80%) were detected. In tank D, three algal phyla (*Bacillariophyta*, 1.93%; *Chlorophyta*, 94.69%; *Rhodophyta*, 6.05%), three fungal phyla (*Ascomycota*, 0.21%; *Basidiomycota*, 0.02%; *Chytridiomycota*, 2.46%), and four zooplankton/other related phyla (*Bryozoa*, 0.04%; *Ciliophora*, 0.30%; *Nematoda*, 0.25%; *Perkinsozoa*, 0.06%) were detected. Finally, in tank E, three algal phyla (*Bacillariophyta*, 12.17%; *Chlorophyta*, 6.86%; *Rhodophyta*, 0.63%), one fungal phylum (*Ascomycota*, 0.04%), and six zooplankton/other related phyla (*Bryozoa*, 0.03%; *Cercozoa*, 0.06%; *Ciliophora*, 59.59%; *Gastrotricha*, 5.45%; *Nematoda*, 10.76%; *Perkinsozoa*, 4.41%) were detected. Among the detected phyla, *Bacillariophyta* (tank E, 12.17%), *Chlorophyta* (tank A, 25.99%; tank B, 94.78%; tank D, 94.69%; tank E, 6.05%), *Ciliophora* (tank A, 32.13%; tank E, 59.59%), *Discosea* (tank C, 5.22%), *Gastrotricha* (tank A, 40.44%; tank C, 79.14%; tank E, 5.45%), *Nematoda* (tank E, 10.76%), and *Rotifera* (tank C, 8.80%) had a relative abundance >5% in the eukaryotic microbial community. Specific trends were observed in the eukaryotic microbial community composition of each sample. For example, the communities in tanks B and D, algal group-dominated, were dominant in *Chlorophyta* (>94% relative abundance). Alternatively, the communities in tanks A, C, and tank E, which were zooplankton/other group-dominated, consisted of three or more phyla, including algal phyla, with a relative abundance >5%.

In the prokaryotic microbial communities of the biofloc, 13 bacterial phyla (*Acidobacteria, Actinobacteria*, *Bacteroidetes*, *Balneolaeota, Candidatus Melainabacteria, Chlamydiae*, *Chloroflexi*, *Cyanobacteria*, *Firmicutes, Planctomycetes, Proteobacteria*, *Tenericutes*, and *Verrucomicrobia*) were detected (Figure 4B). In tank A, the prokaryotic microbial community consisted of ten bacterial phyla (*Actinobacteria*, 1.49%; *Bacteroidetes*, 44.52%; *Candidatus Melainabacteria*, 0.03%; *Chlamydiae*, 0.18%; *Chloroflexi*, 0.49%; *Cyanobacteria*, 3.74%; *Firmicutes*, 0.07%; *Planctomycetes*, 0.75%; *Proteobacteria*, 48.72%; *Verrucomicrobia*, 0.01%). In tank B, it comprised nine bacterial phyla (*Actinobacteria*, 0.87%; *Bacteroidetes*, 76.30%; *Balneolaeota*, 3.44%; *Chlamydiae*, 0.01%; *Cyanobacteria*, 0.87%; *Firmicutes*, 0.01%; *Proteobacteria*, 18.10%; *Tenericutes*, 0.02%; *Verrucomicrobia*, 0.38%). In tank C, it consisted of ten bacterial phyla (*Acidobacteria*, less than 0.01%; *Actinobacteria*, 4.83%; *Bacteroidetes*, 65.70%; Chlamydiae, 0.09%; *Chloroflexi*, 2.28%; *Cyanobacteria*, 0.02%; *Firmicutes*, 0.22%; *Planctomycetes*, 2.45%; *Proteobacteria*, 22.86%; *Verrucomicrobia*, 1.55%). In tank D, it comprised nine bacterial phyla (*Actinobacteria*, 1.99%; *Bacteroidetes*, 33.74%; *Balneolaeota*, 0.07%; *Chlamydiae*, 0.02%; *Chloroflexi*, 10.35%; *Cyanobacteria*, 11.76%; *Planctomycetes*, 0.34%; *Proteobacteria*, 41.05%; *Verrucomicrobia*, 0.68%). Lastly, in tank E, it consisted of 11 bacterial phyla (*Acidobacteria*, 0.03%; *Actinobacteria*, 3.46%; *Bacteroidetes*, 42.18%; *Balneolaeota*, 0.03%; *Chlamydiae*, 0.01%; *Chloroflexi*, 2.47%; *Cyanobacteria*, 28.46%; *Firmicutes*, 0.34%; *Planctomycetes*, 1.94%; *Proteobacteria*, 19.20%; *Verrucomicrobia*, 1.88%). Among the 13 bacterial phyla detected, *Bacteroidetes* (tank A, 44.52%; tank B, 76.30%; tank C, 65.70%; tank D, 33.74%; tank E, 42.18%), *Chloroflexi* (tank D, 10.35%), *Cyanobacteria* (tank D, 11.76%; tank E, 28.46%), and *Proteobacteria* (tank A, 48.72%; tank B, 18.10%; tank C, 22.86%; tank D, 41.05%; tank E, 19.20%) all had a relative abundance >5% in the prokaryotic microbial community. In all samples, *Bacteroidetes* and *Proteobacteria* tended to be dominant, whereas *Chloroflexi* and *Cyanobacteria* appeared at high levels, with *Bacteroidetes* and *Proteobacteria*, in tanks D and E only.

In this study, the composition of the microbial community was determined at the phylum level. The zooplankton/other groups in the studied shrimp farms were mostly composed of *Ciliophora*, *Discosea*, *Gastrotricha*, and *Nematoda*, which are phyla containing algal and bacterial predators (Nosek and Bereczky, 1983; Poinar, 2010; Riera and Todaro, 2012; Santoferrara et al., 2017; Fisher et al., 2019; Lotonin and Smirnov, 2020). The algal group mostly consisted of *Bacillariophyta* (diatom) and *Chlorophyta* (green algae) containing suspended microalgae (Car et al., 2020; Mohseni et al., 2020; Tahir et al., 2020). The bacterial group was mainly composed of *Bacteroidetes*, *Chloroflexi*, *Cyanobacteria*, and *Proteobacteria* (Kawaichi et al., 2013; Chen et al., 2019; Liu et al., 2019; Zammit, 2019; Tanay et al., 2020). Species associated with nitrogen-related metabolism may be present among these phyla (Kawaichi et al., 2013; Chen et al., 2019; Liu et al., 2019; Tanay et al., 2020). Additionally, *Cyanobacteria* is involved in nitrogen-related metabolism through functions such as photosynthesis and nitrogen fixation (Zammit, 2019). In the studied microbial communities, algal and bacterial groups would therefore be consumed by zooplankton and related organisms (Abakari et al., 2020; Sgnaulin et al., 2020), while the algal and bacterial groups would compete for nitrogen (Liu et al., 2019; Urakawa et al., 2019; D’Silva and Kyndt, 2020). Given that the bacterial group was more abundant than the algal group under conditions in which the zooplankton/other groups were dominant (e.g., tanks A, C, and E), predation by the zooplankton and related organisms has a greater impact on the algal group than on the bacterial group (Fisher et al., 2019). Concurrently, the algal group thrived relative to the bacterial group under conditions in which the influence of the zooplankton/other group was weak (e.g., tanks B and C). Thus, shrimp farms using biofloc systems are more suitable environments for algae to bloom than for bacteria to thrive (Cirri and Pohnert, 2019; González-Camejo et al., 2020). In summary, this study suggests that nitrogen-related metabolism in shrimp farms using biofloc technology is mainly carried out by algae. Still, when predation by zooplankton is predominant, nitrogen-related metabolism is instead conducted mainly by bacteria. This phenomenon is expected to occur because the zooplankton and related organisms in shrimp farms affect algae more than bacteria.

### 3.4 Expected roles and functionality of major microbial species in shrimp farms with biofloc systems

Among the microbial species that mainly constituted the microbial communities of the studied shrimp farms, the three algal species were diatom (*P. panduriforme*) and green algae (*T. marina* and *Nannochloris sp*.) types, which are photosynthetic floating microalgae (Car et al., 2020; Mohseni et al., 2020; Tahir et al., 2020). They absorb nitrogen sources, such as ammonia, and synthesize proteins through metabolism, including photosynthesis (El-Sheekh et al., 1994; González-Camejo et al., 2020). Therefore, the algal species in these shrimp farms are expected to play significant roles in the nitrogen cycle and as producers in the ecosystems of the microbial community and shrimp farm (Peace et al., 2014; González-Camejo et al., 2020).

From the taxonomic identification results, the detected species (from the phylum level to the species level) are summarized in Supplementary Tables S1, S2. Among the detected species (91 eukaryotic species and 276 prokaryotic species), the species for which relative abundance was >5% in each of the eukaryotic and prokaryotic microbial communities are listed in Table 2. In tank A, *Nannochloris sp*. (25.26%), *Strombidium guangdongense* (31.09%), and *Halichaetonotus aculifer* (40.44%) were most abundant in the eukaryotic microbial community. In comparison, *Donghicola eburneus* (8.43%), *Muricauda lutimaris* (17.73%), and *Geoalkalibacter subterraneus* (21.51%) were highly prevalent in the prokaryotic microbial community. Tetraselmis marina (90.28%) dominated the eukaryotic microbial community in tank B, while *Polaribacter marinivivus* (56.47%) dominated the prokaryotic microbial community. In tank C, *H. aculifer* (79.11%) dominated the eukaryotic microbial community, while *Lutimonas halocynthiae* (27.66%) was the most prevalent in the prokaryotic microbial community. In tank D, *Nannochloris sp*. (94.69%) dominated the eukaryotic microbial community, whereas *D. eburneus* (22.65%) was the most abundant in the prokaryotic microbial community. Finally, in tank E, *Psammodictyon panduriforme* (11.74%) and *Acineta tuberosa* (59.01%) were particularly abundant in the eukaryotic microbial community. In comparison, *Tenacibaculum aestuarii* (20.91%) and *Loriellopsis cavernicola* (27.90%) were the most prevalent in the prokaryotic microbial community.

**TABLE 2:** Taxonomy and relative abundance of predominant strains in eukaryotic and prokaryotic microbial communities from the five tanks at the studied *Litopenaeus vannamei* shrimp farms.

Community	Organism	Taxonomy					Relative abundance (%)				
		Phylum	Class	Order	Family	Species	Tank A	Tank B	Tank C	Tank D	Tank E
Eukaryotic	Algae	Bacillariophyta	Bacillariophyceae	Bacillariales	Bacillariaceae	*Psammodictyon panduriforme*	0.10	0.00	0.00	0.00	11.74
		Chlorophyta	Chlorodendrophyceae	Chlorodendrales	Chlorodendraceae	*Tetraselmis marina*	0.21	90.28	0.00	0.00	0.00
		Chlorophyta	Trebouxiophyceae	Chlorellales	Chlorellaceae	*Nannochloris* sp.	25.26	0.43	3.23	94.69	5.92
		*Ciliophora*	Phyllopharyngea	Endogenida	Acinetidae	*Acineta tuberosa*	0.00	0.00	0.00	0.00	59.01
	Zoo plankton/Other	*Ciliophora*	Spirotrichea	__	Strombidiidae	*Strombidium guangdongense*	31.09	1.71	0.00	0.00	0.00
		*Discosea*	__	Stygamoebida	__	*Vermistella sp*.	0.00	0.00	5.22	0.00	0.00
		*Gastrotricha*	__	Chaetonotida	Chaetonotidae	*Halichaetonotus aculifer*	40.44	0.00	79.11	0.00	5.35
		Rotifera	Monogononta	Ploima	Brachionidae	*Brachionus plicatilis*	0.14	0.00	8.80	0.00	0.00
Total abundance of marked species in the eukaryotic microbial community							97.24	92.42	97.33	94.76	87.25
Prokaryotic	Bacteria	*Bacteroidetes*	Flavobacteriia	Flavobacteriales	Flavobacteriaceae	*Formosa haliotis*	2.31	0.00	4.70	4.30	5.45
		*Bacteroidetes*	Flavobacteriia	Flavobacteriales	Flavobacteriaceae	*Lutimonas halocynthiae*	0.00	0.00	27.66	0.00	0.00
		*Bacteroidetes*	Flavobacteriia	Flavobacteriales	Flavobacteriaceae	*Lutimonas saemankumensis*	0.15	0.00	0.11	14.74	3.78
		*Bacteroidetes*	Flavobacteriia	Flavobacteriales	Flavobacteriaceae	*Muricauda lutimaris*	17.73	0.05	2.29	0.01	0.76
		*Bacteroidetes*	Flavobacteriia	Flavobacteriales	Flavobacteriaceae	*Polaribacter marinivivus*	0.04	56.47	0.53	0.08	0.75
		*Bacteroidetes*	Flavobacteriia	Flavobacteriales	Flavobacteriaceae	*Tenacibaculum aestuarii*	5.24	0.05	2.72	2.36	20.91
		*Chloroflexi*	Caldilineae	Caldilineales	Caldilineaceae	*Litorilinea aerophila*	0.46	0.00	0.14	10.32	1.97
		*Cyanobacteria*	__	Nostocales	Symphyonemataceae	*Loriellopsis cavernicola*	0.80	0.01	0.02	11.59	27.90
		*Proteobacteria*	Alpha*Proteobacteria*	Rhodobacterales	Rhodobacteraceae	*Donghicola eburneus*	8.43	2.79	0.70	22.65	0.64
		*Proteobacteria*	Alpha*Proteobacteria*	Rhodobacterales	Rhodobacteraceae	*Ruegeria marisrubri*	7.39	0.07	1.25	0.20	3.86
		*Proteobacteria*	Delta*Proteobacteria*	Desulfuromonadales	Geobacteraceae	*Geoalkalibacter subterraneus*	21.51	0.03	0.00	0.00	0.25
		*Proteobacteria*	Gamma*Proteobacteria*	Alteromonadales	Alteromonadaceae	*Mangrovitalea sediminis*	0.00	0.00	0.00	6.71	0.45
Total abundance of marked species in the prokaryotic microbial community							64.06	59.47	40.12	72.96	66.72

The microbial species detected in at least one of the five samples are indicated. Unclassified taxonomic names (phylum, class, order, family, and species) are replaced using underlining (__).

The five major zooplankton/other related species in the studied microbial communities included unicellular amoebae (*Discosea*: *Vermistella sp*.) and multicellular zooplankton (*Ciliophora*: *A. tuberosa* and *S. guangdongense*; *Gastrotricha*: *H. aculifer; Rotifera: Brachionus plicatilis*) (Nosek and Bereczky, 1983; Poinar, 2010; Santoferrara et al., 2017; Fisher et al., 2019; Lotonin and Smirnov, 2020). These algal and bacterial predators consume decaying organic matter (Nosek and Bereczky, 1983; Riera and Todaro, 2012; Santoferrara et al., 2017; Fisher et al., 2019; Lotonin and Smirnov, 2020); however, they unexpected to be primarily involved in the nitrogen cycle or organic compound synthesis because they do not photosynthesize and use inorganic-type nitrogen sources (Caron, 1991; Li Y. et al., 2020). Therefore, zooplankton/other related species will play a role in nitrogen cycle as mediators that convert organic nitrogen into inorganic nitrogen or transfer organic nitrogen to other organisms (Caron, 1991; Li Y. et al., 2020). These processes occur because the zooplankton or related species are primary consumers (Caron, 1991; Li Y. et al., 2020). Previous studies on the influence of consumers on producers suggest that the features of the consumption process can affect the selection of producer species and change the species diversity of communities (Li Y. et al., 2020; Ling and Moreau, 2020; Williams et al., 2020). In this study, it was confirmed that algal group atrophy and relative bacterial group prosperity occurred in environments dominated by the zooplankton/other groups and that the algal group conversely prospered in environments in which the zooplankton/other groups were weak. Therefore, zooplankton and related species in shrimp farms using biofloc technology will directly and indirectly affect the nitrogen cycle as consumers (Caron, 1991; Li Y. et al., 2020).

In the eukaryotic microbial communities of all samples, 1–4 dominant species accounted for 82.02%–96.79% of the total abundance (tank A, 96.79%; tank B, 90.28%; tank C, 93.13%; tank D, 94.69%; tank E, 82.02%), whereas 1–5 dominant species accounted for 27.66%–66.01% of the total abundance in the prokaryotic microbial community (tank A, 60.30%; tank B, 56.47%; tank C, 27.66%; tank D, 66.01%; tank E, 54.26%). There were examples of dominant species with a relative abundance >90% in the eukaryotic microbial community: *T. marina* (tank B, 90.28%) and *Nannochloris sp*. (tank D, 94.69%). Others dominated with lower relative abundances >50%: *A. tuberosa* (tank E, 59.01%) and *H. aculifer* (tank C, 79.11%). However, in the prokaryotic microbial community, all highly abundant species except *P. marinivivus* (tank B, 56.47%) had a relative abundance <30%. These results show that the eukaryotic microbial communities were populated by extremely dominant species, whereas species with relatively lower abundance were prevalent in prokaryotic microbial communities.

The bacterial group in the studied microbial communities contained 12 major species. These bacterial species have been detected and reported in sources involved in the elimination of substances such as ammonia and nitrates (Kawaichi et al., 2013; Chen et al., 2019; Liu et al., 2019; Zammit, 2019; Tanay et al., 2020), e.g., sewage treatment plants, and have been studied for their involvement in nitrogen-related metabolic processes such as nitrification and denitrification (Liu et al., 2019; Abakari et al., 2020; D’Silva and Kyndt, 2020). Additionally, the *Cyanobacteria L. cavernicola* can photosynthesize and is expected to function similarly to members of the algal group (Zammit, 2019). Furthermore, several previous studies have shown that the synthesis of proteins by bacteria is involved in the ammonia and nitrogen cycles and in nitrate-related metabolism (Liu et al., 2019; Tanay et al., 2020). Given these diverse metabolic functions, members of the bacterial group would not be limited to ecological roles as decomposers but may also serve as producers (Riley and Gordon, 1999; Denef and Banfield, 2012). Given that prokaryotic microbial communities in the studied shrimp farms had higher species richness and diversity than the eukaryotic microbial communities, the major species of the bacterial group were diverse, and their nitrogen-related metabolism and ecological roles are likely to be multifarious (Liu et al., 2019; Abakari et al., 2020; Tanay et al., 2020).

In result, in shrimp farms using biofloc technology, microbial communities differ in terms of the major species in each organism group. Still, they seem to have common features in terms of their function and ecological role (Abakari et al., 2020). However, as the bacterial group consists of various major species, relatively clear differences exist among microbial communities with the function of bacterial species (Abakari et al., 2020). Generally, the studied microbial communities had similarities in nitrogen-related metabolism and ecological roles, despite observed differences in environmental factors and the composition of microbial communities. Thus, this study proposes that various metabolic processes are conducted by various species from the bacterial group, which plays many roles in shrimp farms with biofloc systems.

### 3.5 Ecological communication between microbial groups and the nitrogen cycle in shrimp farms using biofloc technology

**FIGURE 5:**
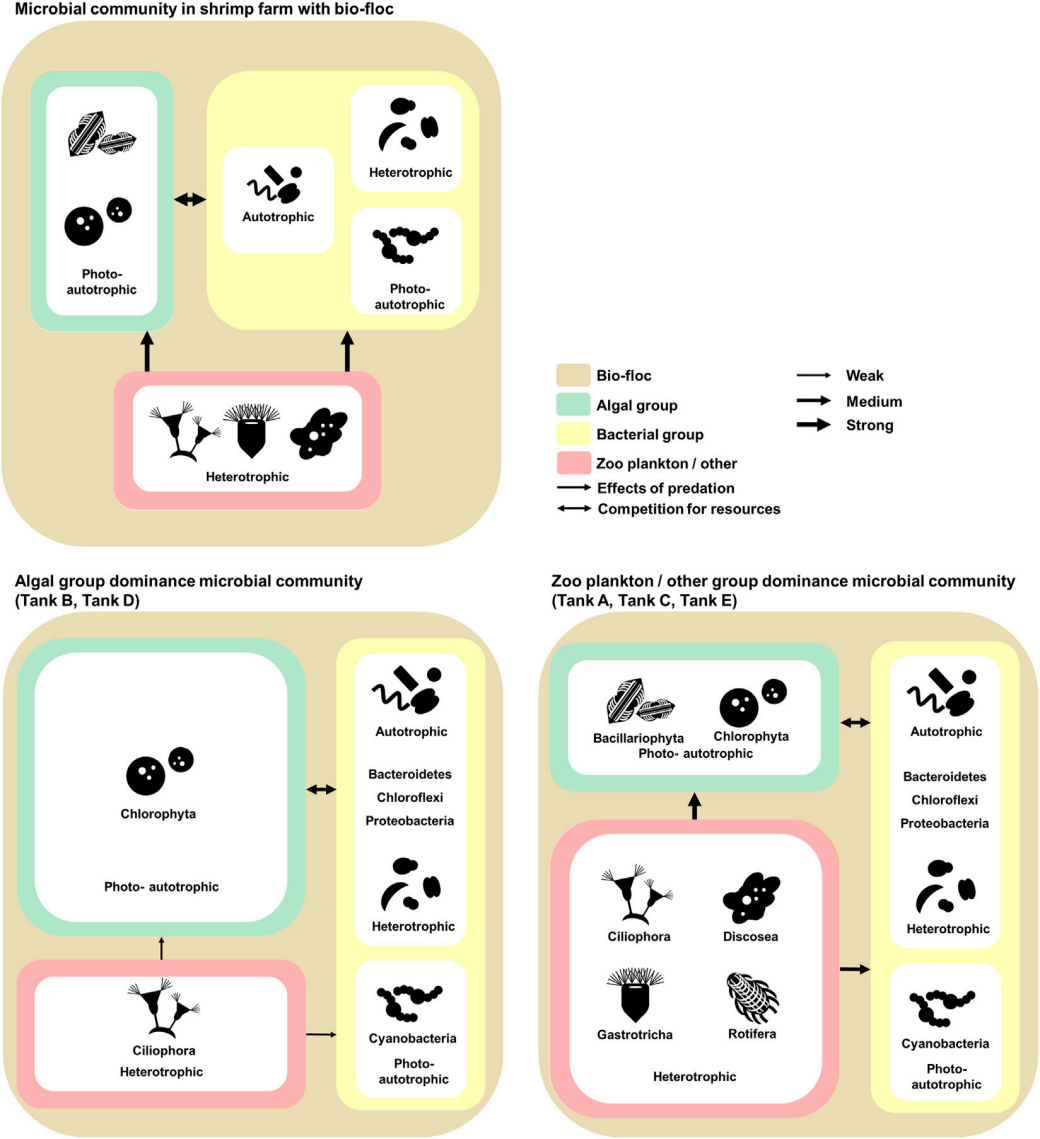
The investigated model of relationships and communications in the microbial communities of *Litopenaeus vannamei* shrimp farms with biofloc technology. The microbial communities coincide with biofloc technology. The environmental features of the aquatic environment in the shrimp farms. The data underlying all the diagrams indicated in this figure can be found in Table 2 and Supplementary Tables S1, S2.

**FIGURE 6:**
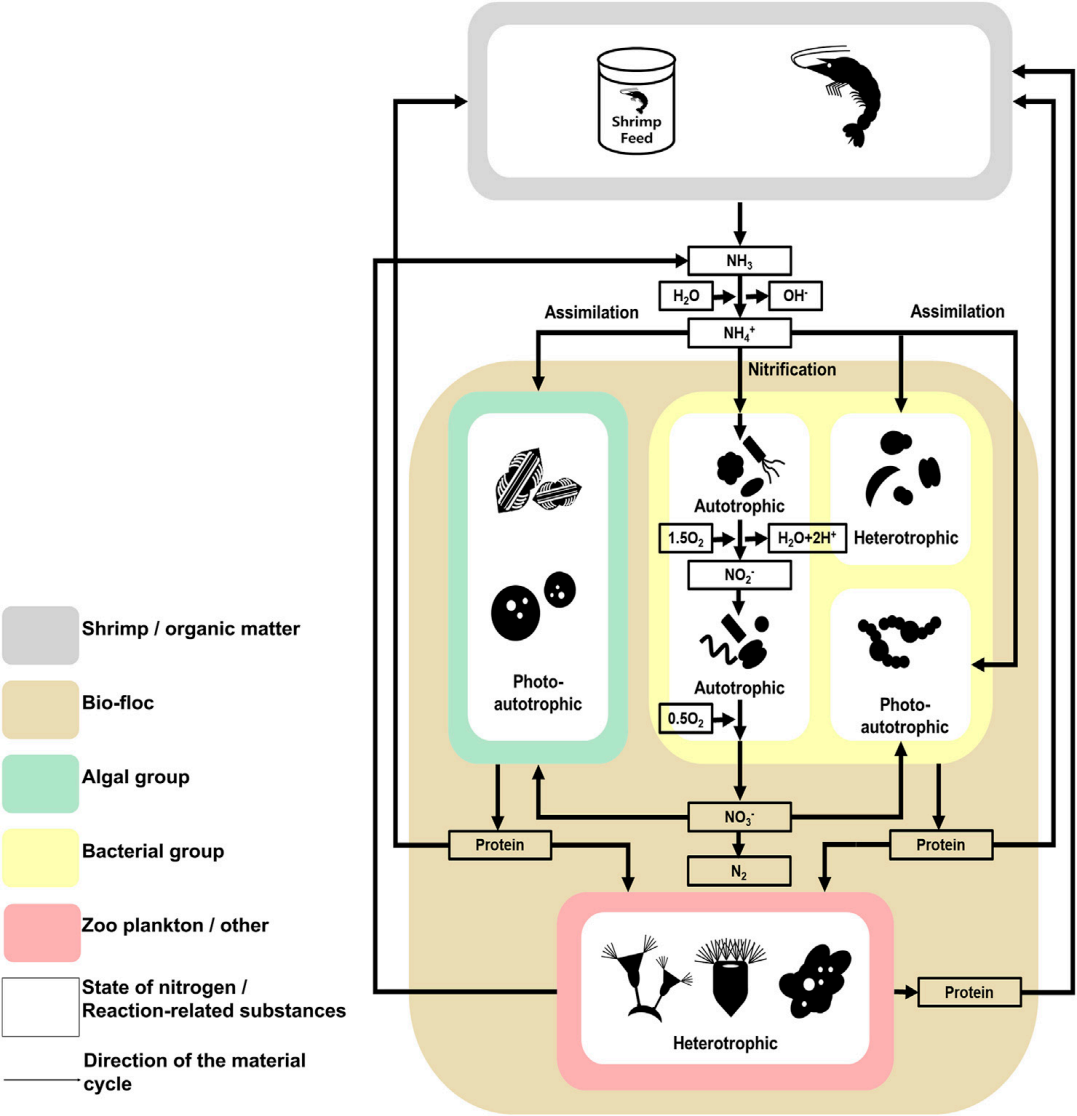
The investigated model of the nitrogen cycle in the microbial communities found in the studied *Litopenaeus vannamei* shrimp farm with biofloc technology. The nitrogen cycle followed in the studied *Litopenaeus vannamei* shrimp farms. *Litopenaeus vannamei* shrimp farms studied in biofloc technology. Physicochemical Characteristics of the Shrimp farm aquatic environment. The data underlying all the diagrams indicated in this figure can be found in Table 1 and Supplementary Tables S1, S2.

Referring to previous research and the results, the ecological communication and nitrogen cycle of the microbial communities in shrimp farms using biofloc systems was illustrated (Figures 5, 6). In these microbial communities, the algal group comprised photoautotrophic organisms, and the zooplankton/other groups comprised heterotrophic organisms (Browdy et al., 2012; Loureiro et al., 2012). Alternatively, the bacterial groups consisted of photoautotrophic, autotrophic, and heterotrophic organisms (Miranda-Baeza et al., 2017; Tanay et al., 2020). Considering the ecological features of the members, competition for limited nitrogen resources would likely occur between the algal and bacterial groups (Figure 5) (Riley and Gordon, 1999; González-Camejo et al., 2020). Additionally, the zooplankton/other groups would affect the algal and bacterial group through predation (Figure 5) (Fisher et al., 2019). This model is supported by the structure of the microbial communities in the investigated samples. For example, the algal group dominates the microbial communities of tanks B and D, which had a relatively low abundance of the zooplankton/other groups. Alternatively, in the microbial communities of tanks A, C, and E, which had a relatively large abundance of the zooplankton/other groups, the bacterial groups were more dominant than the algal groups. Thus, the zooplankton/other groups in shrimp farms using biofloc systems has a stronger influence on the algal groups than the bacterial groups (Fisher et al., 2019; Stoecker and Pierson, 2019). Additionally, under conditions in which the influence of predation is excluded, the algal groups outcompete the bacterial groups because it is more adept at using nitrogen resources than the bacterial groups (Zhu et al., 2019).

The nitrogen cycle also indicates why algal groups are more dominant than bacterial groups when the selection pressure due to predation is low (Figure 6) (Riley and Gordon, 1999; González-Camejo et al., 2020). According to previous studies, algae can use several nitrogen sources, including ammonia and nitric acid, and the abundance of these sources is related to the phenomenon of algal bloom (Zhu et al., 2019; Yao et al., 2020). In contrast, although species associated with nitrogen in the bacterial group use several metabolic processes (e.g., nitrification and assimilation), the type of nitrogen source available to a particular species is limited, or the metabolism process is not actively conducted (Jordan et al., 2005). Additionally, the prosperity of the bacterial group is more dependent on organic carbon sources than on nitrogen (Liu et al., 2019; Tanay et al., 2020). In shrimp farms, a certain amount of nitrogen can be maintained through the nitrogen cycle (Abakari et al., 2020). Alternatively, organic carbon sources depend on externally derived organic carbon sources, except for those produced by photosynthetic organisms. The carbon cycle is not fully part of the shrimp farm ecosystem (Crab et al., 2012). Therefore, the microbial communities of shrimp farms depend on the nitrogen cycle and external organic carbon sources. In conclusion, the effects of predation and the nitrogen cycle strongly affect the composition of microbial communities in shrimp farms in biofloc systems.

## 4 Conclusions

In biofloc systems of the investigated shrimp farms, the prokaryotic microbial communities tended to have higher species richness and diversity than the eukaryotic microbial communities. However, the eukaryotic microbial communities were more abundant and dominant than their prokaryotic counterparts. Overall, the eukaryotic microbial communities were dominated by algae and zooplankton/other related organisms. Therefore, there seem to be unique microbial communities in individual shrimp farms using biofloc technology, but commonalities and patterns appear in these microbial communities. Particularly, the structures of the microbial communities in these shrimp farms depend on the effects of predation by zooplankton and other related organisms; this structure is likely to be shown in the characteristics of the nitrogen cycle in the biofloc system. In this study, we provided many insights into the microbial communities existing in shrimp farms using biofloc technology on the southern coast of the Korean Peninsula. The results provided nitrogen circulation and nitrogen-related metabolism by microorganisms, such as bacteria and algae.

## Data Availability

The datasets presented in this study can be found in online repositories. The names of the repository/repositories and accession number(s) can be found below: National Center for Biotechnology Information (NCBI) BioProject database under accession number PRJNA710740.

## References

[B1] Abakari G. Luo G. Kombat E. O. (2020). Dynamics of nitrogenous compounds and their control in biofloc technology (BFT) systems: A review. Aquacult Fish. 6 (5), 441–447. 10.1016/j.aaf.2020.05.005

[B2] Arantes R. Schveitzer R. Seiffert W. Q. Lapa K. R. Vinatea L. (2017). Nutrient discharge, sludge quantity and characteristics in biofloc shrimp culture using two methods of carbohydrate fertilization. Aquac. Eng. 76, 1–8. 10.1016/j.aquaeng.2016.11.002

[B3] Arnold S. J. Coman F. E. Jackson C. J. Groves S. A. (2009). High-intensity, zero water-exchange production of juvenile tiger shrimp, *Penaeus monodon*: An evaluation of artificial substrates and stocking density. Aquaculture 293, 42–48. 10.1016/j.aquaculture.2009.03.049

[B4] Avnimelech Y. (1999). Carbon/nitrogen ratio as a control element in aquaculture systems. Aquaculture 176, 227–235. 10.1016/s0044-8486(99)00085-x

[B5] Bakar N. S. A. Nasir N. M. Lananan F. Hamid S. H. A. Lam S. S. Jusoh A. (2015). Optimization of C/N ratios for nutrient removal in aquaculture system culturing African catfish, (*Clarias gariepinus*) utilizing Bioflocs Technology. Int. Biodeterior. Biodegrad. 102, 100–106. 10.1016/j.ibiod.2015.04.001

[B6] Bokulich N. A. Subramanian S. Faith J. J. Gevers D. Gordon J. I. Knight R. (2013). Quality-filtering vastly improves diversity estimates from Illumina amplicon sequencing. Nat. Methods 10 (1), 57–59. 10.1038/nmeth.2276 23202435 PMC3531572

[B7] Bossier P. Ekasari J. (2017). Biofloc technology application in aquaculture to support sustainable development goals. Microb. Biotechnol. 10 (5), 1012–1016. 10.1111/1751-7915.12836 28941177 PMC5609229

[B8] Browdy C. L. Ray A. J. Leffler J. W. Avnimelech Y. (2012). Biofloc-based aquaculture systems. Aquac. Prod. Syst. U. S. Willey Blackwell 12, 278–307. 10.1002/9781118250105

[B9] Car A. Hafner D. Ljubimir S. Dupčić Radić I. Bobanović-Ćolić S. Jasprica N. (2020). Colonization of bacteria and diatoms on an artificial substrate in a marine lake (eastern Adriatic Sea, NE Mediterranean). Acta Bot. Croat. 79 (2), 212–227. 10.37427/botcro-2020-028

[B10] Cardona E. Lorgeoux B. Geffroy C. Richard P. Saulnier D. Gueguen Y. (2015). Relative contribution of natural productivity and compound feed to tissue growth in blue shrimp (*Litopenaeus stylirostris*) reared in biofloc: assessment by C and N stable isotope ratios and effect on key digestive enzymes. Aquaculture 448, 288–297. 10.1016/j.aquaculture.2015.05.035

[B11] Caron D. A. (1991). “Evolving role of protozoa in aquatic nutrient cycles,” in Protozoa and their role in marine processes (Springer), 387–415.

[B12] Chen X. Luo G. Meng H. Tan H. (2019). Effect of the particle size on the ammonia removal rate and the bacterial community composition of bioflocs. Aquac. Eng. 86, 102001. 10.1016/j.aquaeng.2019.102001

[B13] Cirri E. Pohnert G. (2019). Algae− bacteria interactions that balance the planktonic microbiome. New Phytol. 223 (1), 100–106. 10.1111/nph.15765 30825329

[B14] Claassen S. du Toit E. Kaba M. Moodley C. Zar H. J. Nicol M. P. (2013). A comparison of the efficiency of five different commercial DNA extraction kits for extraction of DNA from faecal samples. J. Microbiol. Methods 94 (2), 103–110. 10.1016/j.mimet.2013.05.008 23684993 PMC5809576

[B15] Crab R. Defoirdt T. Bossier P. Verstraete W. (2012). Biofloc technology in aquaculture: Beneficial effects and future challenges. Aquaculture 356, 351–356. 10.1016/j.aquaculture.2012.04.046

[B16] Cui H. Ma H. Chen S. Yu J. Xu W. Zhu X. (2020). Mitigating excessive ammonia nitrogen in chicken farm flushing wastewater by mixing strategy for nutrient removal and lipid accumulation in the green alga *Chlorella sorokiniana* . Bioresour. Technol. 303, 122940. 10.1016/j.biortech.2020.122940 32044649

[B17] D’Silva A. Kyndt J. (2020). Bacterial diversity greatly affects ammonia and overall nitrogen levels in aquabioponics bioflocs systems, based on 16S rRNA gene amplicon metagenomics. Appli. Microbiol. 6, 169. 10.35248/2471-9315.20.6.169

[B18] Dai Z. Yu M. Chen H. Zhao H. Huang Y. Su W. (2020). Elevated temperature shifts soil N cycling from microbial immobilization to enhanced mineralization, nitrification and denitrification across global terrestrial ecosystems. Glob. Change Biol. 26 (9), 5267–5276. 10.1111/gcb.15211 32614503

[B19] Dauda A. B. (2020). Biofloc technology: a review on the microbial interactions, operational parameters and implications to disease and health management of cultured aquatic animals. Rev. Aquac. 12 (2), 1193–1210. 10.1111/raq.12379

[B20] Denef V. J. Banfield J. F. (2012). *In situ* evolutionary rate measurements show ecological success of recently emerged bacterial hybrids. Science 336 (6080), 462–466. 10.1126/science.1218389 22539719

[B21] El-Sheekh M. M. Kotkat H. M. Hammouda O. H. (1994). Effect of atrazine herbicide on growth, photosynthesis, protein synthesis, and fatty acid composition in the unicellular green alga *Chlorella kessleri* . Ecotoxicol. Environ. Saf. 29 (3), 349–358. 10.1016/0147-6513(94)90007-8 7534691

[B22] Fisher C. L. Ward C. S. Lane P. D. Kimbrel J. A. Sale K. L. Stuart R. K. (2019). Bacterial communities protect the alga *Microchloropsis salina* from grazing by the rotifer *Brachionus plicatilis* . Algal Res. 40, 101500. 10.1016/j.algal.2019.101500

[B23] Gaitán-Angulo M. Viloria A. Diaz J. C. Laverde H. Ramírez M. C. (2016). Economic evaluation, for the production of white cachama and nilotíca Tilapia (black) in cultivation systems bioflocs. Int. J. Control. Theory Appl. 9 (44), 261–265.

[B24] García-de-la-Fuente R. Cuesta G. Sanchís-Jiménez E. Botella S. Abad M. Fornes F. (2011). Bacteria involved in sulfur amendment oxidation and acidification processes of alkaline ‘alperujo’compost. Bioresour. Technol. 102 (2), 1481–1488. 10.1016/j.biortech.2010.09.103 20970324

[B25] Giordani A. Rodriguez R. P. Sancinetti G. P. Hayashi E. A. Beli E. Brucha G. (2019). Effect of low pH and metal content on microbial community structure in an anaerobic sequencing batch reactor treating acid mine drainage. Min. Eng. 141, 105860. 10.1016/j.mineng.2019.105860

[B26] González-Camejo J. Montero P. Aparicio S. Ruano M. Borrás L. Seco A. (2020). Nitrite inhibition of microalgae induced by the competition between microalgae and nitrifying bacteria. Water Res. 172, 115499. 10.1016/j.watres.2020.115499 31978839

[B27] Hamidoghli A. Yun H. Shahkar E. Won S. Hong J. Bai S. C. (2018). Optimum dietary protein‐to‐energy ratio for juvenile whiteleg shrimp, *Litopenaeus vannamei*, reared in a biofloc system. Aquac. Res. 49 (5), 1875–1886. 10.1111/are.13643

[B28] Heck K. L. Jr van Belle G. Simberloff D. (1975). Explicit calculation of the rarefaction diversity measurement and the determination of sufficient sample size. Ecology 56 (6), 1459–1461. 10.2307/1934716

[B29] Huo S. Kong M. Zhu F. Qian J. Huang D. Chen P. (2020). Co-culture of *Chlorella* and wastewater-borne bacteria in vinegar production wastewater: Enhancement of nutrients removal and influence of algal biomass generation. Algal Res. 45, 101744. 10.1016/j.algal.2019.101744

[B30] Jordan F. L. Cantera J. J. L. Fenn M. E. Stein L. Y. (2005). Autotrophic ammonia-oxidizing bacteria contribute minimally to nitrification in a nitrogen-impacted forested ecosystem. Appl. Environ. Microbiol. 71 (1), 197–206. 10.1128/aem.71.1.197-206.2005 15640188 PMC544198

[B31] Kawaichi S. Ito N. Kamikawa R. Sugawara T. Yoshida T. Sako Y. (2013). Ardenticatena maritima gen. nov., sp. nov., a ferric iron-and nitrate-reducing bacterium of the phylum ‘*Chloroflexi*’isolated from an iron-rich coastal hydrothermal field, and description of *Ardenticatenia* classis nov. Int. J. Syst. Evol. Microbiol. 63 (8), 2992–3002. 10.1099/ijs.0.046532-0 23378114

[B32] Kawale H. D. Kishore N. (2019). Production of hydrocarbons from a green algae (*Oscillatoria*) with exploration of its fuel characteristics over different reaction atmospheres. Energy 178, 344–355. 10.1016/j.energy.2019.04.103

[B33] Khanjani M. H. Sharifinia M. (2020). Biofloc technology as a promising tool to improve aquaculture production. Rev. Aquac. 12, raq.12412–1850. 10.1111/raq.12412

[B34] Klindworth A. Pruesse E. Schweer T. Peplies J. Quast C. Horn M. (2013). Evaluation of general 16S ribosomal RNA gene PCR primers for classical and next-generation sequencing-based diversity studies. Nucleic Acids Res. 41 (1), e1. 10.1093/nar/gks808 22933715 PMC3592464

[B35] Kozich J. J. Westcott S. L. Baxter N. T. Highlander S. K. Schloss P. D. (2013). Development of a dual-index sequencing strategy and curation pipeline for analyzing amplicon sequence data on the MiSeq Illumina sequencing platform. Appl. Environ. Microbiol. 79 (17), 5112–5120. 10.1128/aem.01043-13 23793624 PMC3753973

[B36] Lei F. VanderGheynst J. (2000). The effect of microbial inoculation and pH on microbial community structure changes during composting. Process Biochem. 35 (9), 923–929. 10.1016/s0032-9592(99)00155-7

[B37] Li W. Fu L. Niu B. Wu S. Wooley J. (2012). Ultrafast clustering algorithms for metagenomic sequence analysis. Brief. Bioinform. 13 (6), 656–668. 10.1093/bib/bbs035 22772836 PMC3504929

[B38] Li L. Chen Z. Huang Q. (2020). Exogenous γ-aminobutyric acid promotes biomass and astaxanthin production in *Haematococcus pluvialis* . Algal Res. 52, 102089. 10.1016/j.algal.2020.102089

[B39] Li Y. Miao Y. Zhang W. Yang N. Niu L. Zhang H. (2020). Sertraline inhibits top-down forces (predation) in microbial food web and promotes nitrification in sediment. Environ. Pollut. 267, 115580. 10.1016/j.envpol.2020.115580 33254665

[B40] Ling Y.-C. Moreau J. W. (2020). Bacterial predation limits microbial sulfate-reduction in a coastal acid sulfate soil (CASS) ecosystem. Soil Biol. Biochem. 148, 107930. 10.1016/j.soilbio.2020.107930

[B41] Liu W. Tan H. Luo G. Yu Y. Zhang N. Yao M. (2019). Effects of C/N ratio on nitrogen removal with denitrification phase after a nitrification-based biofloc aquaculture cycle. Aquac. Eng. 86, 101994. 10.1016/j.aquaeng.2019.101994

[B42] López-Mondéjar R. Tláskal V. Větrovský T. Štursová M. Toscan R. da Rocha U. N. (2020). Metagenomics and stable isotope probing reveal the complementary contribution of fungal and bacterial communities in the recycling of dead biomass in forest soil. Soil Biol. Biochem. 148, 107875. 10.1016/j.soilbio.2020.107875

[B43] Lotonin K. Smirnov A. (2020). Stygamoeba cauta n. sp.(*Amoebozoa, discosea*)–a new brackish-water species from nivå bay (baltic sea, the sound). Eur. J. Protistol. 72, 125660. 10.1016/j.ejop.2019.125660 31835237

[B44] Loureiro C. K. Wasielesky W. Abreu P. C. (2012). The use of protozoan, rotifers and nematodes as live food for shrimp raised in BFT system. Atlântica 34 (1), 5–12. 10.5088/atl.2012.34.1.5

[B45] Luo G. Chen X. Tan J. Abakari G. Tan H. (2020). Effects of carbohydrate addition strategy and biofloc levels on the establishment of nitrification in biofloc technology aquaculture systems. Aquaculture 514, 734441. 10.1016/j.aquaculture.2019.734441

[B46] Luo G. Xu J. Li J. Zheng H. Tan H. Liu W. (2022). Rapid production bioflocs by inoculation and fertilized with different nitrogen and carbon sources. Aquac. Eng. 98, 102262. 10.1016/j.aquaeng.2022.102262

[B47] Meyer M. Kircher M. (2010). Illumina sequencing library preparation for highly multiplexed target capture and sequencing. Cold Spring Harb. Protoc. 2010 (6), pdb. prot5448. 10.1101/pdb.prot5448 20516186

[B48] Mickalide H. Kuehn S. (2019). Higher-order interaction between species inhibits bacterial invasion of a phototroph-predator microbial community. Cell Syst. 9 (6), 521–533.e10. 10.1016/j.cels.2019.11.004 31838145

[B49] Miranda‐Baeza A. Mariscal‐López M. A. López‐Elías J. A. Rivas‐Vega M. E. Emerenciano M. Sánchez‐Romero A. (2017). Effect of inoculation of the *Cyanobacteria* Oscillatoria sp. on tilapia biofloc culture. Aquac. Res. 48 (9), 4725–4734. 10.1111/are.13294

[B50] Miranda‐Baeza A. Nolasco‐López M. Rivas‐Vega M. E. Huerta‐Rábago J. A. Martínez‐Córdova L. R. Martínez‐Porchas M. (2020). Short‐term effect of the inoculation of probiotics in mature bioflocs: Water quality parameters and abundance of heterotrophic and ammonia‐oxidizing bacteria. Aquac. Res. 51 (1), 255–264. 10.1111/are.14371

[B51] Mohseni A. Kube M. Fan L. Roddick F. A. (2020). Potential of Chlorella vulgaris and Nannochloropsis salina for nutrient and organic matter removal from municipal wastewater reverse osmosis concentrate. Environ. Sci. Pollut. Res. 27 (21), 26905–26914. 10.1007/s11356-020-09103-6 32382902

[B52] Nosek J. Bereczky M. C. (1983). “Structural investigations of periphytic protozoan communities in three layers of the Danube River,” in Periphyton of freshwater ecosystems (Springer), 55–58.

[B53] Peace A. Wang H. Kuang Y. (2014). Dynamics of a producer–grazer model incorporating the effects of excess food nutrient content on grazer’s growth. Bull. Math. Biol. 76 (9), 2175–2197. 10.1007/s11538-014-0006-z 25124765

[B54] Pilgrim A. Gray F. Weller R. Belling C. (1970). Synthesis of microbial protein from ammonia in the sheep's rumen and the proportion of dietary nitrogen converted into microbial nitrogen. Br. J. Nutr. 24 (2), 589–598. 10.1079/bjn19700057 5452709

[B55] Poinar G. O. Jr (2010). “Nematoda and nematomorpha,” in Ecology and classification of North American freshwater invertebrates. Editors Thorp J. H. Covich A. P.. 3rd edition (Elsevier), 237–276.

[B56] Putra I. Effendi I. Lukistyowati I. Tang U. M. Fauzi M. Suharman I. (2020). Effect of different biofloc starters on ammonia, nitrate, and nitrite concentrations in the cultured tilapia *Oreochromis niloticus* system. F1000Res. 9, 293. 10.12688/f1000research.22977.3 32509278 PMC7241270

[B57] Ray A. J. Shuler A. J. Leffler J. W. Browdy C. L. (2009). “Microbial ecology and management of biofloc systems,” in The rising tide: Proceedings of the special session on sustainable shrimp farming. Editors Browdy C. L. Jory D. E. (Baton Rouge, LA, USA: World Aquaculture Society), 231–242.

[B58] Riera R. Todaro M. A. (2012). Check list of gastrotrichs from the canary islands. Rev. Acad. Canar. Cienc. 24, 81–88.

[B59] Riley M. A. Gordon D. M. (1999). The ecological role of bacteriocins in bacterial competition. Trends Microbiol. 7 (3), 129–133. 10.1016/s0966-842x(99)01459-6 10203843

[B60] Sanjit L. Bhatt D. (2005). How relevant are the concepts of species diversity and species richness? J. Biosci. 30 (5), 557–560. 10.1007/bf02703552 16388126

[B61] Santi I. Tsiola A. Dimitriou P. D. Fodelianakis S. Kasapidis P. Papageorgiou N. (2019). Prokaryotic and eukaryotic microbial community responses to N and P nutrient addition in oligotrophic Mediterranean coastal waters: Novel insights from DNA metabarcoding and network analysis. Mar. Environ. Res. 150, 104752. 10.1016/j.marenvres.2019.104752 31326679

[B62] Santoferrara L. F. Alder V. V. McManus G. B. (2017). Phylogeny, classification and diversity of choreotrichia and oligotrichia (*Ciliophora*, spirotrichea). Mol. Phylogenet. Evol. 112, 12–22. 10.1016/j.ympev.2017.03.010 28286224

[B63] Schloss P. D. Westcott S. L. Ryabin T. Hall J. R. Hartmann M. Hollister E. B. (2009). Introducing mothur: open-source, platform-independent, community-supported software for describing and comparing microbial communities. Appl. Environ. Microbiol. 75 (23), 7537–7541. 10.1128/aem.01541-09 19801464 PMC2786419

[B64] Sgnaulin T. Durigon E. G. Pinho S. M. Jerônimo G. T. de Alcantara Lopes D. L. Emerenciano M. G. C. (2020). Nutrition of Genetically Improved Farmed Tilapia (GIFT) in biofloc technology system: Optimization of digestible protein and digestible energy levels during nursery phase. Aquaculture 521, 734998. 10.1016/j.aquaculture.2020.734998

[B65] Shiraiwa Y. Goyal A. Tolbert N. (1993). Alkalization of the medium by unicellular green algae during uptake dissolved inorganic carbon. Plant Cell Physiol. 34 (5), 649–657. 10.1093/oxfordjournals.pcp.a078467

[B66] Stoeck T. Bass D. Nebel M. Christen R. Jones M. D. Breiner H. W. (2010). Multiple marker parallel tag environmental DNA sequencing reveals a highly complex eukaryotic community in marine anoxic water. Mol. Ecol. 19, 21–31. 10.1111/j.1365-294x.2009.04480.x 20331767

[B67] Stoecker D. Pierson J. (2019). Predation on protozoa: its importance to zooplankton revisited. J. Plankton Res. 41 (4), 367–373. 10.1093/plankt/fbz027

[B68] Sturm M. Schroeder C. Bauer P. (2016). SeqPurge: highly-sensitive adapter trimming for paired-end NGS data. BMC Bioinforma. 17 (1), 208. 10.1186/s12859-016-1069-7 PMC4862148 27161244

[B69] Tahir A. Rukminasari N. Yaqin K. Lukman M. (2020). Increasing CO_2_ concentration impact upon nutrient absorption and removal efficiency of supra intensive shrimp pond wastewater by marine microalgae *Tetraselmis chui* . Int. J. Phytorem. 23 (1), 64–71. 10.1080/15226514.2020.1791051 32662344

[B70] Takai K. (2019). The nitrogen cycle: A large, fast, and mystifying cycle. Microbes Environ. 34 (3), 223–225. 10.1264/jsme2.me3403rh 31554776 PMC6759337

[B71] Tanay D. D. Abella T. T. Cruz E. M. V. Sace C. F. Fajardo L. J. Velasco R. R. (2020). Microbial community response to carbon-nitrogen ratio manipulation in biofloc culture. Philipp. J. Fish. 27 (2), 193–207. 10.31398/tpjf/27.2.2019a0014

[B73] Taw N. (2014). “Biofloc as biosecurity: A possible solution in preventing shrimp disease,” in Proceedings of the World Aquaculture, Adelaide, Australia, 7–11.

[B74] Timmons M. B. Summerfelt S. T. Vinci B. J. (1998). Review of circular tank technology and management. Aquac. Eng. 18 (1), 51–69. 10.1016/s0144-8609(98)00023-5

[B75] Urakawa H. Rajan S. Feeney M. E. Sobecky P. A. Mortazavi B. (2019). Ecological response of nitrification to oil spills and its impact on the nitrogen cycle. Environ. Microbiol. 21 (1), 18–33. 10.1111/1462-2920.14391 30136386

[B76] Van Den Hende S. Vervaeren H. Desmet S. Boon N. (2011). Bioflocculation of microalgae and bacteria combined with flue gas to improve sewage treatment. N. Biotechnol. 29 (1), 23–31. 10.1016/j.nbt.2011.04.009 21565287

[B77] Vilani F. G. Schveitzer R. Da Fonseca Arantes R. Do Nascimento Vieira F. Do Espirito Santo C. M. Seiffert W. Q. (2016). Strategies for water preparation in a biofloc system: Effects of carbon source and fertilization dose on water quality and shrimp performance. Aquac. Eng. 74, 70–75. 10.1016/j.aquaeng.2016.06.002

[B78] Vo A. T. Jedlicka J. A. (2014). Protocols for metagenomic DNA extraction and Illumina amplicon library preparation for faecal and swab samples. Mol. Ecol. Resour. 14 (6), 1183–1197. 10.1111/1755-0998.12269 24774752

[B79] Wang H. Qi B. Jiang X. Jiang Y. Yang H. Xiao Y. (2019). Microalgal interstrains differences in algal-bacterial biofloc formation during liquid digestate treatment. Bioresour. Technol. 289, 121741. 10.1016/j.biortech.2019.121741 31323710

[B80] Wei G. Shan D. Li G. Li X. Tian R. He J. (2020). Prokaryotic communities vary with floc size in a biofloc-technology based aquaculture system. Aquaculture 529, 735632. 10.1016/j.aquaculture.2020.735632

[B81] Wei Y.-F. Wang A.-L. Liao S.-A. (2020). Effect of different carbon sources on microbial community structure and composition of *ex-situ* biofloc formation. Aquaculture 515, 734492. 10.1016/j.aquaculture.2019.734492

[B82] Williams C. L. Thomas B. J. McEwan N. R. Rees Stevens P. Creevey C. J. Huws S. A. (2020). Rumen protozoa play a significant role in fungal predation and plant carbohydrate breakdown. Front. Microbiol. 11, 720. 10.3389/fmicb.2020.00720 32411103 PMC7200989

[B83] Yao X. Zhang Y. Zhang L. Zhu G. Qin B. Zhou Y. (2020). Emerging role of dissolved organic nitrogen in supporting algal bloom persistence in Lake Taihu, China: Emphasis on internal transformations. Sci. Total Environ. 736, 139497. 10.1016/j.scitotenv.2020.139497 32502780

[B84] Zammit G. (2019). Phototrophic biofilm communities and adaptation to growth on ancient archaeological surfaces. Ann. Microbiol. 69 (10), 1047–1058. 10.1007/s13213-019-01471-w

[B85] Zhang Z. Schwartz S. Wagner L. Miller W. (2000). A greedy algorithm for aligning DNA sequences. J. Comput. Biol. 7 (1-2), 203–214. 10.1089/10665270050081478 10890397

[B86] Zhang X.-Y. Peng D.-C. Wan Q. Ju K. Wang B.-B. Pei L.-Y. (2020). Changing the nutrient source from ammonia to nitrate: Effects on heterotrophic bacterial growth in wastewater. Pol. J. Environ. Stud. 29 (2), 1473–1482. 10.15244/pjoes/109219

[B87] Zhu L. Li S. Hu T. Nugroho Y. K. Yin Z. Hu D. (2019). Effects of nitrogen source heterogeneity on nutrient removal and biodiesel production of mono-and mix-cultured microalgae. Energy Convers. Manag. 201, 112144. 10.1016/j.enconman.2019.112144

