# Supplementary material for: The microbial communities (bacteria, algae, zooplankton, and fungi) improved biofloc technology including the nitrogen-related material cycle in Litopenaeus vannamei farms
Source: Front Bioeng Biotechnol. 2022 Nov 23;10:883522. doi: 10.3389/fbioe.2022.883522 (PMC9727081; doi:10.3389/fbioe.2022.883522)
Supplement: Supplementary file 1 [file DataSheet1.docx]

**Supplemental Information**

**The Microbial Communities (Bacteria, Algae, Zooplankton, and Fungi) Improved Biofloc Technology Including the Nitrogen-Related Material Cycle in *Litopenaeus vannamei* Farms**

**Hyun-Sik Yun^1,†^, Dong-Hyun Kim^2,†^, Jong-Guk Kim^2,4,*^, Young-Saeng Kim^3,*^**, **Ho-Sung Yoon^1,4,5,*^**

^1^ Department of Biology, College of Natural Sciences, Kyungpook National University, Daegu 41566, Republic of Korea

^2^ School of Applied Biosciences, Kyungpook National University, Daegu, 37224, Republic of Korea

^3^ Research Institute of Ulleung-do & Dok-do, Kyungpook National University, Daegu 41566, Republic of Korea

^4^ School of Life Sciences and Biotechnology, BK21 Plus KNU Creative BioResearch Group, Kyungpook National University, Daegu, 41566, Republic of Korea

^5^ Advanced Bio-Resource Research Center, Kyungpook National University, Daegu 41566, Republic of Korea

**^†^** These authors contributed equally to this work

*** Correspondence:**

Tel.: +82-53-950-5348

Young-Saeng Kim: kyslhh1228@hanmail.net

Jong-Guk Kim: [kimjg@knu.ac.kr](mailto:kimjg@knu.ac.kr)

Ho-Sung Yoon: hsy@knu.ac.kr

**TABLE S1** Taxonomy and frequency of species in the eukaryotic microbial communities from the five tanks at the studied *Litopenaeus vannamei* shrimp farms.

| Taxonomy | | | | | | Frequency | | | | |
| --- | --- | --- | --- | --- | --- | --- | --- | --- | --- | --- |
| Kingdom | Phylum | Class | Order | Family | Species | Tank A | Tank B | Tank C | Tank D | Tank E |
| Eukaryota | Apicomplexa | __ | __ | __ | uncultured *apicomplexan* | 0 | 0 | 19 | 0 | 0 |
| Eukaryota | Arthropoda | Malacostraca | Decapoda | Penaeidae | *Penaeus vannamei* | 0 | 0 | 12 | 0 | 0 |
| Eukaryota | Ascomycota | __ | __ | __ | uncultured *Ascomycota* | 0 | 0 | 31 | 0 | 0 |
| Eukaryota | Ascomycota | Saccharomycetes | Saccharomycetales | Debaryomycetaceae | *Schwanniomyces etchellsii* | 0 | 0 | 0 | 64 | 0 |
| Eukaryota | Ascomycota | Saccharomycetes | Saccharomycetales | Phaffomycetaceae | *Wickerhamomyces subpelliculosus* | 0 | 0 | 0 | 8 | 0 |
| Eukaryota | Ascomycota | Saccharomycetes | Saccharomycetales | Saccharomycetaceae | *Saccharomyces cerevisiae* | 40 | 913 | 57 | 58 | 9 |
| Eukaryota | Bacillariophyta | __ | __ | __ | uncultured *diatom* | 0 | 41 | 0 | 0 | 0 |
| Eukaryota | Bacillariophyta | Bacillariophyceae | Bacillariales | Bacillariaceae | *Cylindrotheca closterium* | 0 | 224 | 0 | 0 | 0 |
| Eukaryota | Bacillariophyta | Bacillariophyceae | Bacillariales | Bacillariaceae | *Nitzschia amphibia* | 0 | 0 | 3 | 0 | 0 |
| Eukaryota | Bacillariophyta | Bacillariophyceae | Bacillariales | Bacillariaceae | *Nitzschia capitellata* | 0 | 44 | 0 | 0 | 0 |
| Eukaryota | Bacillariophyta | Bacillariophyceae | Bacillariales | Bacillariaceae | *Nitzschia* sp. | 6 | 0 | 0 | 0 | 17 |
| Eukaryota | Bacillariophyta | Bacillariophyceae | Bacillariales | Bacillariaceae | *Psammodictyon panduriforme* | 23 | 0 | 0 | 0 | 2866 |
| Eukaryota | Bacillariophyta | Bacillariophyceae | Bacillariales | Bacillariaceae | *Tryblionella gaoana* | 0 | 0 | 0 | 0 | 3 |
| Eukaryota | Bacillariophyta | Bacillariophyceae | Mastogloiales | Achnanthaceae | *Planothidium caputium* | 0 | 0 | 8 | 0 | 0 |
| Eukaryota | Bacillariophyta | Bacillariophyceae | Naviculales | Amphipleuraceae | *Halamphora subtropica* | 0 | 0 | 0 | 0 | 36 |
| Eukaryota | Bacillariophyta | Bacillariophyceae | Naviculales | Naviculaceae | *Navicula perminuta* | 0 | 48 | 2 | 0 | 16 |
| Eukaryota | Bacillariophyta | Bacillariophyceae | Thalassiophysales | Catenulaceae | *Amphora coffeiformis* | 0 | 0 | 0 | 0 | 9 |
| Eukaryota | Bacillariophyta | Bacillariophyceae | Thalassiophysales | Catenulaceae | *Amphora copulata* | 0 | 0 | 7 | 0 | 0 |
| Eukaryota | Bacillariophyta | Coscinodiscophyceae | Thalassiosirales | Thalassiosiraceae | *Minidiscus comicus* | 33 | 0 | 0 | 0 | 0 |
| Eukaryota | Bacillariophyta | Coscinodiscophyceae | Thalassiosirales | Thalassiosiraceae | *Minidiscus variabilis* | 6 | 0 | 5 | 5 | 0 |
| Eukaryota | Bacillariophyta | Fragilariophyceae | Fragilariales | Staurosiraceae | *Nanofrustulum shiloi* | 0 | 0 | 17 | 0 | 5 |
| Eukaryota | Bacillariophyta | Mediophyceae | Cymatosirales | Cymatosiraceae | *Minutocellus* sp. | 4 | 0 | 0 | 1192 | 18 |
| Eukaryota | Bacillariophyta | Mediophyceae | Hemiaulales | Hemiaulaceae | *Cerataulina pelagica* | 0 | 463 | 0 | 0 | 0 |
| Eukaryota | Basidiomycota | Agaricomycetes | Polyporales | Polyporaceae | *Trametes versicolor* | 0 | 5 | 0 | 0 | 0 |
| Eukaryota | Basidiomycota | Agaricostilbomycetes | Agaricostilbales | Agaricostilbaceae | *Sterigmatomyces elviae* | 0 | 0 | 0 | 11 | 0 |
| Eukaryota | Basidiomycota | Tremellomycetes | Filobasidiales | Filobasidiaceae | *Filobasidium stepposum* | 0 | 6 | 1 | 0 | 0 |
| Eukaryota | Bryozoa | Gymnolaemata | Cheilostomatida | Adeonidae | *Reptadeonella brasiliensis* | 0 | 0 | 8 | 23 | 7 |
| Eukaryota | Cercozoa | __ | __ | __ | *Cercozoa* sp. | 0 | 0 | 124 | 0 | 1 |
| Eukaryota | Cercozoa | __ | __ | __ | *Cercozoan amoeba* | 0 | 0 | 0 | 0 | 14 |
| Eukaryota | Cercozoa | __ | __ | __ | uncultured *Cercozoa* | 0 | 0 | 54 | 0 | 0 |
| Eukaryota | Cercozoa | __ | Glissomonadida | __ | uncultured *Glissomonadida* | 0 | 0 | 23 | 0 | 0 |
| Eukaryota | Chlorophyta | __ | __ | __ | *Chlorophyta* sp. | 0 | 0 | 1 | 0 | 6 |
| Eukaryota | Chlorophyta | Chlorodendrophyceae | Chlorodendrales | Chlorodendraceae | *Tetraselmis marina* | 49 | 64034 | 0 | 0 | 0 |
| Eukaryota | Chlorophyta | Chlorodendrophyceae | Chlorodendrales | Chlorodendraceae | *Tetraselmis* sp. | 19 | 879 | 0 | 0 | 0 |
| Eukaryota | Chlorophyta | Chlorophyceae | Chlamydomonadales | Chlamydomonadaceae | *Chlamydomonas applanata* | 0 | 0 | 5 | 0 | 0 |
| Eukaryota | Chlorophyta | Chlorophyceae | Chlamydomonadales | Chlamydomonadaceae | *Chlamydomonas concordia* | 0 | 0 | 0 | 0 | 5 |
| Eukaryota | Chlorophyta | Chlorophyceae | Chlamydomonadales | Chlamydomonadaceae | *Chlamydomonas oviformis* | 0 | 0 | 3 | 0 | 0 |
| Eukaryota | Chlorophyta | Chlorophyceae | Chlamydomonadales | Chlamydomonadaceae | *Chlamydomonas pila* | 0 | 0 | 3 | 0 | 0 |
| Eukaryota | Chlorophyta | Chlorophyceae | Chlamydomonadales | Chlamydomonadaceae | *Chlamydomonas raudensis* | 0 | 22 | 0 | 0 | 0 |
| Eukaryota | Chlorophyta | Chlorophyceae | Chlamydomonadales | Chlorococcaceae | *Characium* sp. | 0 | 0 | 4 | 0 | 0 |
| Eukaryota | Chlorophyta | Chlorophyceae | Sphaeropleales | Scenedesmaceae | *Desmodesmus armatus* | 0 | 0 | 175 | 0 | 0 |
| Eukaryota | Chlorophyta | Chlorophyceae | Sphaeropleales | Scenedesmaceae | *Scenedesmus* sp. | 100 | 0 | 62 | 0 | 0 |
| Eukaryota | Chlorophyta | Trebouxiophyceae | __ | __ | *Chloroidium* sp. | 0 | 0 | 28 | 0 | 0 |
| Eukaryota | Chlorophyta | Trebouxiophyceae | __ | __ | *Chloroidium viscosum* | 0 | 0 | 1 | 0 | 7 |
| Eukaryota | Chlorophyta | Trebouxiophyceae | __ | __ | *Jaagichlorella luteoviridis* | 0 | 0 | 0 | 0 | 10 |
| Eukaryota | Chlorophyta | Trebouxiophyceae | __ | __ | *Polulichloris yunnanensis* | 0 | 0 | 15 | 0 | 3 |
| Eukaryota | Chlorophyta | Trebouxiophyceae | Chlorellales | Chlorellaceae | *Nannochloris* sp. | 5914 | 307 | 2028 | 58796 | 1446 |
| Eukaryota | Chlorophyta | Trebouxiophyceae | Trebouxiales | Trebouxiaceae | *Dictyochloropsis asterochloroides* | 0 | 0 | 5 | 0 | 0 |
| Eukaryota | Chlorophyta | Ulvophyceae | Ulotrichales | Ulotrichaceae | *Capsosiphon fulvescens* | 0 | 0 | 3 | 0 | 0 |
| Eukaryota | Chlorophyta | Ulvophyceae | Ulvales | Ulvaceae | *Ulva prolifera* | 4 | 1982 | 1 | 0 | 0 |
| Eukaryota | Chytridiomycota | __ | __ | __ | uncultured *Chytridiomycota* | 0 | 0 | 0 | 1526 | 0 |
| Eukaryota | Ciliophora | __ | __ | __ | *uncultured ciliate* | 0 | 0 | 15 | 0 | 0 |
| Eukaryota | Ciliophora | Litostomatea | Haptorida | Pseudotrachelocercidae | *Pseudotrachelocerca trepida* | 0 | 0 | 110 | 0 | 0 |
| Eukaryota | Ciliophora | Oligohymenophorea | Philasterida | Cinetochilidae | *Cinetochilum ovale* | 0 | 0 | 0 | 6 | 0 |
| Eukaryota | Ciliophora | Oligohymenophorea | Philasterida | Pseudocohnilembidae | *Pseudocohnilembus persalinus* | 0 | 0 | 0 | 0 | 107 |
| Eukaryota | Ciliophora | Oligohymenophorea | Philasterida | Uronematidae | *Uronema nigricans* | 0 | 0 | 0 | 177 | 0 |
| Eukaryota | Ciliophora | Oligohymenophorea | Pleuronematida | Cyclidiidae | *Cyclidium glaucoma* | 112 | 62 | 0 | 0 | 0 |
| Eukaryota | Ciliophora | Oligohymenophorea | Sessilida | Vorticellidae | *Pseudovorticella punctata* | 32 | 0 | 0 | 0 | 0 |
| Eukaryota | Ciliophora | Oligohymenophorea | Sessilida | Zoothamniidae | *Zoothamnium* sp. | 0 | 0 | 10 | 1 | 0 |
| Eukaryota | Ciliophora | Phyllopharyngea | Dysteriida | Dysteriidae | *Dysteriidae* sp. | 52 | 0 | 24 | 0 | 5 |
| Eukaryota | Ciliophora | Phyllopharyngea | Dysteriida | Dysteriidae | *Dysteria nabia* | 0 | 0 | 94 | 0 | 0 |
| Eukaryota | Ciliophora | Phyllopharyngea | Dysteriida | Hartmannulidae | *Aegyriana oliva* | 0 | 0 | 11 | 0 | 0 |
| Eukaryota | Ciliophora | Phyllopharyngea | Endogenida | Acinetidae | *Acineta tuberosa* | 0 | 0 | 0 | 0 | 14401 |
| Eukaryota | Ciliophora | Spirotrichea | __ | Strombidiidae | *Parallelostrombidium paralatum* | 0 | 0 | 100 | 0 | 0 |
| Eukaryota | Ciliophora | Spirotrichea | __ | Strombidiidae | *Strombidium guangdongense* | 7279 | 1214 | 0 | 0 | 0 |
| Eukaryota | Ciliophora | Spirotrichea | Euplotida | Aspidiscidae | *Aspidisca hexeris* | 49 | 0 | 25 | 0 | 30 |
| Eukaryota | Ciliophora | Spirotrichea | Euplotida | Euplotidae | *Euplotes parabalteatus* | 0 | 0 | 0 | 3 | 1 |
| Eukaryota | Ciliophora | Spirotrichea | Euplotida | Uronychiidae | *Diophrys oligothrix* | 0 | 72 | 181 | 0 | 0 |
| Eukaryota | Ciliophora | Spirotrichea | Euplotida | Uronychiidae | *Diophrys parappendiculata* | 0 | 296 | 0 | 0 | 0 |
| Eukaryota | Ciliophora | Spirotrichea | Sporadotrichida | Gonostomatidae | *Paragonostomoides xianicum* | 0 | 0 | 50 | 0 | 0 |
| Eukaryota | Ciliophora | Spirotrichea | Urostylida | Holostichidae | *Holosticha diademata* | 0 | 221 | 0 | 0 | 0 |
| Eukaryota | Cryptomycota | __ | __ | __ | uncultured *Cryptomycota* | 0 | 0 | 55 | 0 | 0 |
| Eukaryota | Discosea | __ | Stygamoebida | __ | *Vermistella* sp. | 0 | 0 | 3279 | 0 | 0 |
| Eukaryota | Gastrotricha | __ | _haetonotida | Chaetonotidae | *Aspidiophorus polystictos* | 0 | 0 | 0 | 0 | 25 |
| Eukaryota | Gastrotricha | __ | Chaetonotida | Chaetonotidae | *Halichaetonotus aculifer* | 9468 | 0 | 49656 | 0 | 1305 |
| Eukaryota | Gastrotricha | __ | Chaetonotida | Chaetonotidae | *Heterolepidoderma* sp. | 0 | 0 | 4 | 0 | 0 |
| Eukaryota | Gastrotricha | __ | Chaetonotida | Chaetonotidae | *Polymerurus rhomboides* | 0 | 0 | 15 | 0 | 0 |
| Eukaryota | Imbricatea | __ | Euglyphida | Cyphoderiidae | *Cyphoderia littoralis* | 0 | 0 | 14 | 0 | 0 |
| Eukaryota | Nematoda | __ | __ | __ | *Nematoda environmental* | 8 | 0 | 0 | 0 | 67 |
| Eukaryota | Nematoda | Chromadorea | Monhysterida | Monhysteridae | *Monhysteridae* sp. | 0 | 0 | 0 | 0 | 15 |
| Eukaryota | Nematoda | Chromadorea | Monhysterida | Monhysteridae | *Diplolaimella dievengatensis* | 0 | 0 | 16 | 158 | 25 |
| Eukaryota | Nematoda | Chromadorea | Monhysterida | Monhysteridae | *Monhystrella parvella* | 132 | 0 | 65 | 0 | 348 |
| Eukaryota | Nematoda | Chromadorea | Rhabditida | Rhabditidae | *Litoditis sp.* | 0 | 0 | 113 | 0 | 1155 |
| Eukaryota | Nematoda | Chromadorea | Rhabditida | Rhabditidae | *Oscheius* sp. | 15 | 0 | 0 | 0 | 1015 |
| Eukaryota | Perkinsozoa | __ | Perkinsida | Perkinsidae | *Parvilucifera rostrata* | 0 | 0 | 606 | 43 | 1076 |
| Eukaryota | Rhodophyta | Bangiophyceae | Bangiales | Bangiaceae | *Pyropia* sp. | 37 | 0 | 100 | 24 | 0 |
| Eukaryota | Rhodophyta | Stylonematophyceae | Stylonematales | Stylonemataceae | *Rhodosorus marinus* | 0 | 0 | 0 | 0 | 153 |
| Eukaryota | Rotifera | Monogononta | Ploima | Brachionidae | *Brachionus plicatilis* | 32 | 0 | 5523 | 0 | 98 |
| Eukaryota | Tubulinea | Elardia | Leptomyxida | Flabellulidae | *Flabellula pomeranica* | 0 | 93 | 0 | 0 | 102 |
| Total frequency | | | | | | 23414 | 70926 | 62771 | 62095 | 24406 |

The microbial species detected in at least one of the five samples are shown. Unclassified taxonomic names (phylum, class, order, family, and species) are replaced using underlining (__).

**TABLE S2** Taxonomy and frequency of species in the prokaryotic microbial community of the five tanks from the shrimp (*Litopenaeus vannamei*) farms.

| Taxonomy | | | | | | Frequency | | | | |
| --- | --- | --- | --- | --- | --- | --- | --- | --- | --- | --- |
| Kingdom | Phylum | Class | Order | Family | Species | Tank A | Tank B | Tank C | Tank D | Tank E |
| Bacteria | Acidobacteria | Acidobacteriia | Bryobacterales | Bryobacteraceae | *Paludibaculum fermentans* | 0 | 0 | 1 | 0 | 6 |
| Bacteria | Actinobacteria | Acidimicrobiia | Acidimicrobiales | Acidimicrobiaceae | *Aciditerrimonas ferrireducens* | 0 | 0 | 20 | 0 | 0 |
| Bacteria | Actinobacteria | Acidimicrobiia | Acidimicrobiales | Iamiaceae | *Aquihabitans daechungensis* | 8 | 0 | 51 | 12 | 34 |
| Bacteria | Actinobacteria | Acidimicrobiia | Acidimicrobiales | Iamiaceae | *Iamia majanohamensis* | 5 | 0 | 371 | 8 | 29 |
| Bacteria | Actinobacteria | Acidimicrobiia | Acidimicrobiales | Ilumatobacteraceae | *Ilumatobacter fluminis* | 22 | 1 | 37 | 10 | 13 |
| Bacteria | Actinobacteria | Acidimicrobiia | Acidimicrobiales | Ilumatobacteraceae | *Ilumatobacter nonamiensis* | 0 | 0 | 10 | 0 | 0 |
| Bacteria | Actinobacteria | Actinobacteria | Corynebacteriales | Mycobacteriaceae | *Mycobacterium aquaticum* | 281 | 0 | 112 | 57 | 369 |
| Bacteria | Actinobacteria | Actinobacteria | Corynebacteriales | Mycobacteriaceae | *Mycolicibacterium mucogenicum* | 0 | 0 | 8 | 1 | 0 |
| Bacteria | Actinobacteria | Actinobacteria | Geodermatophilales | Geodermatophilaceae | *Blastococcus jejuensis* | 8 | 1 | 23 | 41 | 131 |
| Bacteria | Actinobacteria | Actinobacteria | Geodermatophilales | Geodermatophilaceae | *Blastococcus saxobsidens* | 1 | 0 | 4 | 1 | 13 |
| Bacteria | Actinobacteria | Actinobacteria | Geodermatophilales | Geodermatophilaceae | *Geodermatophilus normandii* | 0 | 0 | 13 | 21 | 1 |
| Bacteria | Actinobacteria | Actinobacteria | Micrococcales | Demequinaceae | *Demequina activiva* | 1 | 0 | 34 | 8 | 1 |
| Bacteria | Actinobacteria | Actinobacteria | Micrococcales | Demequinaceae | *Demequina flava* | 2 | 0 | 188 | 234 | 49 |
| Bacteria | Actinobacteria | Actinobacteria | Micrococcales | Intrasporangiaceae | *Tetrasphaera jenkinsii* | 2 | 0 | 0 | 2 | 0 |
| Bacteria | Actinobacteria | Actinobacteria | Micrococcales | Microbacteriaceae | *Candidatus Limnoluna* | 4 | 360 | 1 | 0 | 3 |
| Bacteria | Actinobacteria | Actinobacteria | Micrococcales | Microbacteriaceae | *Microbacterium awajiense* | 32 | 0 | 82 | 14 | 24 |
| Bacteria | Actinobacteria | Actinobacteria | Propionibacteriales | Nocardioidaceae | *Nocardioides terrae* | 0 | 0 | 1 | 1 | 1 |
| Bacteria | Actinobacteria | Actinobacteria | Pseudonocardiales | Pseudonocardiaceae | *Goodfellowiella coeruleoviolacea* | 0 | 0 | 0 | 36 | 1 |
| Bacteria | Actinobacteria | Actinobacteria | Streptomycetales | Streptomycetaceae | *Streptomyces avicenniae* | 2 | 0 | 270 | 11 | 69 |
| Bacteria | Actinobacteria | Thermoleophilia | Solirubrobacterales | Patulibacteraceae | *Patulibacter medicamentivorans* | 0 | 0 | 3 | 0 | 0 |
| Bacteria | Bacteroidetes | Bacteroidia | Bacteroidales | Bacteroidaceae | *Bacteroides intestinalis* | 0 | 13 | 0 | 0 | 0 |
| Bacteria | Bacteroidetes | Bacteroidia | Marinilabiliales | Marinilabiliaceae | *Carboxylicivirga taeanensis* | 62 | 0 | 11 | 8 | 0 |
| Bacteria | Bacteroidetes | Chitinophagia | Chitinophagales | Chitinophagaceae | *Nemorincola caseinilytica* | 234 | 0 | 17 | 0 | 30 |
| Bacteria | Bacteroidetes | Chitinophagia | Chitinophagales | Chitinophagaceae | *Paracnuella aquatica* | 0 | 0 | 5 | 0 | 0 |
| Bacteria | Bacteroidetes | Chitinophagia | Chitinophagales | Chitinophagaceae | *Rurimicrobium arvi* | 0 | 0 | 3 | 0 | 0 |
| Bacteria | Bacteroidetes | Chitinophagia | Chitinophagales | Chitinophagaceae | *Sediminibacterium salmoneum* | 0 | 3 | 0 | 0 | 0 |
| Bacteria | Bacteroidetes | Cytophagia | Cytophagales | Cyclobacteriaceae | *Algoriphagus formosus* | 0 | 0 | 45 | 0 | 0 |
| Bacteria | Bacteroidetes | Cytophagia | Cytophagales | Cyclobacteriaceae | *Algoriphagus marincola* | 0 | 611 | 6 | 3 | 6 |
| Bacteria | Bacteroidetes | Cytophagia | Cytophagales | Flammeovirgaceae | *Fabibacter halotolerans* | 16 | 0 | 31 | 7 | 0 |
| Bacteria | Bacteroidetes | Cytophagia | Cytophagales | Flammeovirgaceae | *Fabibacter misakiensis* | 0 | 6 | 21 | 0 | 0 |
| Bacteria | Bacteroidetes | Cytophagia | Cytophagales | Flammeovirgaceae | *Fabivirga thermotolerans* | 0 | 0 | 2 | 7 | 6 |
| Bacteria | Bacteroidetes | Cytophagia | Cytophagales | Flammeovirgaceae | *Fulvivirga lutimaris* | 14 | 0 | 120 | 7 | 19 |
| Bacteria | Bacteroidetes | Cytophagia | Cytophagales | Flammeovirgaceae | *Imperialibacter roseus* | 0 | 0 | 13 | 0 | 0 |
| Bacteria | Bacteroidetes | Cytophagia | Cytophagales | Flammeovirgaceae | *Marivirga harenae* | 6 | 0 | 39 | 2 | 0 |
| Bacteria | Bacteroidetes | Cytophagia | Cytophagales | Flammeovirgaceae | *Roseivirga spongicola* | 0 | 7 | 0 | 0 | 0 |
| Bacteria | Bacteroidetes | Cytophagia | Cytophagales | Hymenobacteraceae | *Adhaeribacter aerolatus* | 5 | 0 | 7 | 0 | 163 |
| Bacteria | Bacteroidetes | Cytophagia | Cytophagales | Hymenobacteraceae | *Pontibacter toksunensis* | 0 | 0 | 1 | 1 | 2 |
| Bacteria | Bacteroidetes | Cytophagia | Cytophagales | Microscillaceae | *Microscilla marina* | 0 | 73 | 2 | 0 | 0 |
| Bacteria | Bacteroidetes | Flavobacteriia | Flavobacteriales | Crocinitomicaceae | *Lishizhenia tianjinensis* | 22 | 15 | 7 | 33 | 1 |
| Bacteria | Bacteroidetes | Flavobacteriia | Flavobacteriales | Cryomorphaceae | *Luteibaculum oceani* | 0 | 0 | 195 | 0 | 14 |
| Bacteria | Bacteroidetes | Flavobacteriia | Flavobacteriales | Cryomorphaceae | *Owenweeksia hongkongensis* | 24 | 26 | 547 | 80 | 10 |
| Bacteria | Bacteroidetes | Flavobacteriia | Flavobacteriales | Cryomorphaceae | *Phaeocystidibacter luteus* | 0 | 0 | 3 | 0 | 0 |
| Bacteria | Bacteroidetes | Flavobacteriia | Flavobacteriales | Cryomorphaceae | *Salibacter halophilus* | 0 | 27 | 0 | 0 | 0 |
| Bacteria | Bacteroidetes | Flavobacteriia | Flavobacteriales | Cryomorphaceae | *Vicingus serpentipes* | 251 | 632 | 624 | 172 | 21 |
| Bacteria | Bacteroidetes | Flavobacteriia | Flavobacteriales | Flavobacteriaceae | *Aequorivita aestuarii* | 0 | 0 | 3 | 0 | 0 |
| Bacteria | Bacteroidetes | Flavobacteriia | Flavobacteriales | Flavobacteriaceae | *Aquimarina megaterium* | 0 | 1 | 5 | 0 | 34 |
| Bacteria | Bacteroidetes | Flavobacteriia | Flavobacteriales | Flavobacteriaceae | *Arenibacter algicola* | 0 | 0 | 71 | 0 | 0 |
| Bacteria | Bacteroidetes | Flavobacteriia | Flavobacteriales | Flavobacteriaceae | *Aureisphaera galaxeae* | 149 | 0 | 14 | 6 | 63 |
| Bacteria | Bacteroidetes | Flavobacteriia | Flavobacteriales | Flavobacteriaceae | *Cellulophaga tyrosinoxydans* | 0 | 1956 | 1 | 1 | 207 |
| Bacteria | Bacteroidetes | Flavobacteriia | Flavobacteriales | Flavobacteriaceae | *Confluentibacter lentus* | 96 | 0 | 0 | 1 | 49 |
| Bacteria | Bacteroidetes | Flavobacteriia | Flavobacteriales | Flavobacteriaceae | *Feifantangia zhejiangensis* | 276 | 0 | 1301 | 51 | 4 |
| Bacteria | Bacteroidetes | Flavobacteriia | Flavobacteriales | Flavobacteriaceae | *Flagellimonas flava* | 180 | 0 | 0 | 0 | 12 |
| Bacteria | Bacteroidetes | Flavobacteriia | Flavobacteriales | Flavobacteriaceae | *Flaviramulus ichthyoenteri* | 428 | 0 | 5 | 0 | 10 |
| Bacteria | Bacteroidetes | Flavobacteriia | Flavobacteriales | Flavobacteriaceae | *Flavobacterium haoranii* | 0 | 0 | 4 | 0 | 0 |
| Bacteria | Bacteroidetes | Flavobacteriia | Flavobacteriales | Flavobacteriaceae | *Formosa haliotis* | 569 | 0 | 1196 | 986 | 1163 |
| Bacteria | Bacteroidetes | Flavobacteriia | Flavobacteriales | Flavobacteriaceae | *Gaetbulibacter jejuensis* | 310 | 14 | 361 | 357 | 87 |
| Bacteria | Bacteroidetes | Flavobacteriia | Flavobacteriales | Flavobacteriaceae | *Jejuia pallidilutea* | 0 | 32 | 0 | 0 | 0 |
| Bacteria | Bacteroidetes | Flavobacteriia | Flavobacteriales | Flavobacteriaceae | *Kordia algicida* | 0 | 9 | 0 | 0 | 0 |
| Bacteria | Bacteroidetes | Flavobacteriia | Flavobacteriales | Flavobacteriaceae | *Leptobacterium flavescens* | 0 | 0 | 0 | 0 | 779 |
| Bacteria | Bacteroidetes | Flavobacteriia | Flavobacteriales | Flavobacteriaceae | *Lutibacter litorisediminis* | 4 | 0 | 0 | 0 | 0 |
| Bacteria | Bacteroidetes | Flavobacteriia | Flavobacteriales | Flavobacteriaceae | *Lutimonas halocynthiae* | 0 | 0 | 7031 | 0 | 0 |
| Bacteria | Bacteroidetes | Flavobacteriia | Flavobacteriales | Flavobacteriaceae | *Lutimonas saemankumensis* | 36 | 0 | 27 | 3377 | 805 |
| Bacteria | Bacteroidetes | Flavobacteriia | Flavobacteriales | Flavobacteriaceae | *Maribacter marinus* | 467 | 0 | 1257 | 36 | 39 |
| Bacteria | Bacteroidetes | Flavobacteriia | Flavobacteriales | Flavobacteriaceae | *Muricauda lutimaris* | 4367 | 21 | 583 | 2 | 161 |
| Bacteria | Bacteroidetes | Flavobacteriia | Flavobacteriales | Flavobacteriaceae | *Muricauda olearia* | 436 | 1 | 3 | 0 | 4 |
| Bacteria | Bacteroidetes | Flavobacteriia | Flavobacteriales | Flavobacteriaceae | *Nonlabens aestuariivivens* | 0 | 13 | 0 | 0 | 0 |
| Bacteria | Bacteroidetes | Flavobacteriia | Flavobacteriales | Flavobacteriaceae | *Nonlabens dokdonensis* | 0 | 16 | 0 | 0 | 0 |
| Bacteria | Bacteroidetes | Flavobacteriia | Flavobacteriales | Flavobacteriaceae | *Nonlabens sediminis* | 0 | 1266 | 0 | 0 | 0 |
| Bacteria | Bacteroidetes | Flavobacteriia | Flavobacteriales | Flavobacteriaceae | *Planktosalinus lacus* | 0 | 0 | 0 | 0 | 3 |
| Bacteria | Bacteroidetes | Flavobacteriia | Flavobacteriales | Flavobacteriaceae | *Polaribacter dokdonensis* | 0 | 544 | 0 | 0 | 0 |
| Bacteria | Bacteroidetes | Flavobacteriia | Flavobacteriales | Flavobacteriaceae | *Polaribacter huanghezhanensis* | 0 | 0 | 2 | 0 | 8 |
| Bacteria | Bacteroidetes | Flavobacteriia | Flavobacteriales | Flavobacteriaceae | *Polaribacter lacunae* | 0 | 32 | 0 | 0 | 3 |
| Bacteria | Bacteroidetes | Flavobacteriia | Flavobacteriales | Flavobacteriaceae | *Polaribacter marinivivus* | 9 | 23453 | 136 | 19 | 159 |
| Bacteria | Bacteroidetes | Flavobacteriia | Flavobacteriales | Flavobacteriaceae | *Polaribacter tangerinus* | 0 | 89 | 0 | 0 | 0 |
| Bacteria | Bacteroidetes | Flavobacteriia | Flavobacteriales | Flavobacteriaceae | *Pseudozobellia thermophila* | 12 | 0 | 40 | 0 | 0 |
| Bacteria | Bacteroidetes | Flavobacteriia | Flavobacteriales | Flavobacteriaceae | *Psychroflexus saliphilus* | 0 | 0 | 1 | 1064 | 58 |
| Bacteria | Bacteroidetes | Flavobacteriia | Flavobacteriales | Flavobacteriaceae | *Psychroserpens mesophilus* | 314 | 10 | 0 | 6 | 42 |
| Bacteria | Bacteroidetes | Flavobacteriia | Flavobacteriales | Flavobacteriaceae | *Sabulilitoribacter arenilitoris* | 159 | 0 | 0 | 0 | 0 |
| Bacteria | Bacteroidetes | Flavobacteriia | Flavobacteriales | Flavobacteriaceae | *Salinimicrobium flavum* | 0 | 0 | 0 | 3 | 0 |
| Bacteria | Bacteroidetes | Flavobacteriia | Flavobacteriales | Flavobacteriaceae | *Spongiimonas flava* | 0 | 0 | 34 | 1 | 1 |
| Bacteria | Bacteroidetes | Flavobacteriia | Flavobacteriales | Flavobacteriaceae | *Taeania maliponensis* | 0 | 0 | 235 | 0 | 0 |
| Bacteria | Bacteroidetes | Flavobacteriia | Flavobacteriales | Flavobacteriaceae | *Tamlana agarivorans* | 21 | 0 | 207 | 82 | 42 |
| Bacteria | Bacteroidetes | Flavobacteriia | Flavobacteriales | Flavobacteriaceae | *Tamlana crocina* | 261 | 1 | 88 | 160 | 14 |
| Bacteria | Bacteroidetes | Flavobacteriia | Flavobacteriales | Flavobacteriaceae | *Tenacibaculum aestuarii* | 1291 | 21 | 691 | 542 | 4458 |
| Bacteria | Bacteroidetes | Flavobacteriia | Flavobacteriales | Flavobacteriaceae | *Tenacibaculum caenipelagi* | 0 | 827 | 0 | 0 | 0 |
| Bacteria | Bacteroidetes | Flavobacteriia | Flavobacteriales | Flavobacteriaceae | *Tenacibaculum gallaicum* | 3 | 0 | 0 | 0 | 0 |
| Bacteria | Bacteroidetes | Flavobacteriia | Flavobacteriales | Flavobacteriaceae | *Tenacibaculum xiamenense* | 0 | 331 | 0 | 0 | 0 |
| Bacteria | Bacteroidetes | Flavobacteriia | Flavobacteriales | Flavobacteriaceae | *Winogradskyella aquimaris* | 0 | 9 | 0 | 1 | 0 |
| Bacteria | Bacteroidetes | Flavobacteriia | Flavobacteriales | Flavobacteriaceae | *Winogradskyella crassostreae* | 0 | 0 | 0 | 0 | 3 |
| Bacteria | Bacteroidetes | Flavobacteriia | Flavobacteriales | Flavobacteriaceae | *Winogradskyella flava* | 616 | 36 | 205 | 1 | 88 |
| Bacteria | Bacteroidetes | Flavobacteriia | Flavobacteriales | Flavobacteriaceae | *Winogradskyella wandonensis* | 0 | 11 | 0 | 0 | 0 |
| Bacteria | Bacteroidetes | Flavobacteriia | Flavobacteriales | Flavobacteriaceae | *Yeosuana aromativorans* | 0 | 0 | 186 | 0 | 0 |
| Bacteria | Bacteroidetes | Flavobacteriia | Flavobacteriales | Flavobacteriaceae | *Zhouia amylolytica* | 3 | 0 | 0 | 0 | 1 |
| Bacteria | Bacteroidetes | Flavobacteriia | Flavobacteriales | Flavobacteriaceae | *Zobellia russellii* | 0 | 0 | 559 | 36 | 68 |
| Bacteria | Bacteroidetes | Saprospiria | Saprospirales | Haliscomenobacteraceae | *Phaeodactylibacter luteus* | 13 | 227 | 1 | 177 | 19 |
| Bacteria | Bacteroidetes | Saprospiria | Saprospirales | Haliscomenobacteraceae | *Phaeodactylibacter xiamenensis* | 168 | 1257 | 755 | 114 | 5 |
| Bacteria | Bacteroidetes | Saprospiria | Saprospirales | Haliscomenobacteraceae | *Portibacter lacus* | 0 | 0 | 0 | 109 | 22 |
| Bacteria | Bacteroidetes | Saprospiria | Saprospirales | Lewinellaceae | *Lewinella cohaerens* | 41 | 99 | 0 | 232 | 254 |
| Bacteria | Bacteroidetes | Saprospiria | Saprospirales | Lewinellaceae | *Lewinella nigricans* | 106 | 0 | 0 | 0 | 35 |
| Bacteria | Bacteroidetes | Saprospiria | Saprospirales | Saprospiraceae | *Membranicola marinus* | 0 | 0 | 0 | 48 | 3 |
| Bacteria | Bacteroidetes | Sphingobacteriia | Sphingobacteriales | Sphingobacteriaceae | *Parapedobacter koreensis* | 0 | 0 | 0 | 0 | 19 |
| Bacteria | Balneolaeota | Balneolia | Balneolales | Balneolaceae | *Gracilimonas halophila* | 0 | 1427 | 0 | 0 | 6 |
| Bacteria | Balneolaeota | Balneolia | Balneolales | Balneolaceae | *Rhodohalobacter halophilus* | 0 | 0 | 0 | 17 | 0 |
| Bacteria | Candidatus Melainabacteria | __ | Vampirovibrionales | __ | *Vampirovibrio chlorellavorus* | 7 | 0 | 0 | 0 | 0 |
| Bacteria | Chlamydiae | Chlamydiia | Parachlamydiales | Parachlamydiaceae | *Neochlamydia hartmannellae* | 0 | 0 | 4 | 0 | 0 |
| Bacteria | Chlamydiae | Chlamydiia | Parachlamydiales | Simkaniaceae | *Simkania negevensis* | 0 | 3 | 20 | 6 | 1 |
| Bacteria | Chlamydiae | Chlamydiia | Parachlamydiales | Waddliaceae | *Waddlia chondrophila* | 43 | 0 | 0 | 0 | 1 |
| Bacteria | Chloroflexi | Anaerolineae | Anaerolineales | Anaerolineaceae | *Thermomarinilinea lacunifontana* | 3 | 0 | 0 | 0 | 0 |
| Bacteria | Chloroflexi | Caldilineae | Caldilineales | Caldilineaceae | *Litorilinea aerophila* | 114 | 1 | 36 | 2365 | 420 |
| Bacteria | Chloroflexi | Thermomicrobia | Sphaerobacterales | Sphaerobacteraceae | *Sphaerobacter thermophilus* | 0 | 0 | 100 | 2 | 67 |
| Bacteria | Chloroflexi | Thermomicrobia | Thermomicrobiales | Thermomicrobiaceae | *Thermomicrobium carboxidum* | 4 | 0 | 444 | 5 | 39 |
| Bacteria | Cyanobacteria | __ | Chroococcales | Aphanothecaceae | *Crocosphaera watsonii* | 724 | 135 | 0 | 28 | 66 |
| Bacteria | Cyanobacteria | __ | Nostocales | Calotrichaceae | *Dulcicalothrix necridiiformans* | 0 | 0 | 0 | 0 | 44 |
| Bacteria | Cyanobacteria | __ | Nostocales | Symphyonemataceae | *Loriellopsis cavernicola* | 196 | 5 | 5 | 2657 | 5949 |
| Bacteria | Cyanobacteria | __ | Pleurocapsales | Dermocarpellaceae | *Stanieria cyanosphaera* | 1 | 222 | 0 | 0 | 0 |
| Bacteria | Cyanobacteria | __ | Pleurocapsales | Xenococcaceae | *Foliisarcina bertiogensis* | 0 | 0 | 0 | 10 | 0 |
| Bacteria | Cyanobacteria | __ | Synechococcales | Chamaesiphonaceae | *Chamaesiphon minutus* | 0 | 0 | 0 | 0 | 10 |
| Bacteria | Firmicutes | Bacilli | Bacillales | Staphylococcaceae | *Staphylococcus equorum* | 0 | 0 | 0 | 0 | 3 |
| Bacteria | Firmicutes | Clostridia | Clostridiales | Eubacteriaceae | *Irregularibacter muris* | 17 | 0 | 51 | 0 | 69 |
| Bacteria | Firmicutes | Clostridia | Clostridiales | Lachnospiraceae | *Kineothrix alysoides* | 0 | 4 | 0 | 0 | 0 |
| Bacteria | Firmicutes | Tissierellia | Tissierellales | Tissierellaceae | *Tepidimicrobium xylanilyticum* | 0 | 0 | 4 | 0 | 0 |
| Bacteria | Planctomycetes | Phycisphaerae | Phycisphaerales | Phycisphaeraceae | *Algisphaera agarilytica* | 0 | 0 | 0 | 0 | 16 |
| Bacteria | Planctomycetes | Planctomycetia | Pirellulales | Lacipirellulaceae | *Bythopirellula goksoyri* | 2 | 0 | 53 | 29 | 63 |
| Bacteria | Planctomycetes | Planctomycetia | Pirellulales | Pirellulaceae | *Blastopirellula cremea* | 1 | 0 | 4 | 2 | 1 |
| Bacteria | Planctomycetes | Planctomycetia | Pirellulales | Pirellulaceae | *Mariniblastus fucicola* | 0 | 0 | 2 | 1 | 0 |
| Bacteria | Planctomycetes | Planctomycetia | Pirellulales | Pirellulaceae | *Rhodopirellula baltica* | 19 | 0 | 3 | 3 | 6 |
| Bacteria | Planctomycetes | Planctomycetia | Pirellulales | Pirellulaceae | *Rhodopirellula caenicola* | 0 | 0 | 0 | 0 | 3 |
| Bacteria | Planctomycetes | Planctomycetia | Pirellulales | Pirellulaceae | *Rhodopirellula rosea* | 0 | 0 | 0 | 14 | 0 |
| Bacteria | Planctomycetes | Planctomycetia | Pirellulales | Pirellulaceae | *Roseimaritima ulvae* | 0 | 0 | 10 | 0 | 11 |
| Bacteria | Planctomycetes | Planctomycetia | Planctomycetales | Planctomycetaceae | *Gimesia maris* | 30 | 0 | 3 | 16 | 268 |
| Bacteria | Planctomycetes | Planctomycetia | Planctomycetales | Planctomycetaceae | *Rubinisphaera brasiliensis* | 134 | 0 | 543 | 12 | 39 |
| Bacteria | Planctomycetes | Planctomycetia | Planctomycetales | Planctomycetaceae | *Schlesneria paludicola* | 0 | 0 | 4 | 0 | 6 |
| Bacteria | Proteobacteria | Alphaproteobacteria | Caulobacterales | Caulobacteraceae | *Aquidulcibacter paucihalophilus* | 2 | 0 | 131 | 61 | 49 |
| Bacteria | Proteobacteria | Alphaproteobacteria | Emcibacterales | Emcibacteraceae | *Emcibacter nanhaiensis* | 0 | 0 | 6 | 0 | 0 |
| Bacteria | Proteobacteria | Alphaproteobacteria | Holosporales | Caedimonadaceae | *Caedimonas varicaedens* | 0 | 3 | 0 | 0 | 0 |
| Bacteria | Proteobacteria | Alphaproteobacteria | Parvularculales | Parvularculaceae | *Marinicaulis aureum* | 51 | 0 | 264 | 0 | 23 |
| Bacteria | Proteobacteria | Alphaproteobacteria | Parvularculales | Parvularculaceae | *Parvularcula bermudensis* | 0 | 0 | 54 | 0 | 0 |
| Bacteria | Proteobacteria | Alphaproteobacteria | Parvularculales | Parvularculaceae | *Parvularcula oceani* | 0 | 0 | 0 | 0 | 3 |
| Bacteria | Proteobacteria | Alphaproteobacteria | Pelagibacterales | Pelagibacteraceae | *Candidatus Pelagibacter ubique* | 0 | 3 | 0 | 0 | 1 |
| Bacteria | Proteobacteria | Alphaproteobacteria | Rhizobiales | __ | *Bauldia consociata* | 70 | 0 | 7 | 8 | 46 |
| Bacteria | Proteobacteria | Alphaproteobacteria | Rhizobiales | __ | *Methyloceanibacter caenitepidi* | 0 | 0 | 2 | 17 | 30 |
| Bacteria | Proteobacteria | Alphaproteobacteria | Rhizobiales | Bradyrhizobiaceae | *Bosea robiniae* | 0 | 0 | 5 | 0 | 1 |
| Bacteria | Proteobacteria | Alphaproteobacteria | Rhizobiales | Brucellaceae | *Ochrobactrum intermedium* | 0 | 0 | 0 | 6 | 0 |
| Bacteria | Proteobacteria | Alphaproteobacteria | Rhizobiales | Brucellaceae | *Ochrobactrum oryzae* | 0 | 0 | 6 | 7 | 6 |
| Bacteria | Proteobacteria | Alphaproteobacteria | Rhizobiales | Cohaesibacteraceae | *Cohaesibacter gelatinilyticus* | 5 | 925 | 1 | 1 | 1 |
| Bacteria | Proteobacteria | Alphaproteobacteria | Rhizobiales | Hyphomicrobiaceae | *Maritalea mobilis* | 0 | 31 | 8 | 0 | 1 |
| Bacteria | Proteobacteria | Alphaproteobacteria | Rhizobiales | Phyllobacteriaceae | *Aquamicrobium soli* | 0 | 0 | 0 | 3 | 0 |
| Bacteria | Proteobacteria | Alphaproteobacteria | Rhizobiales | Phyllobacteriaceae | *Hoeflea halophila* | 7 | 0 | 8 | 8 | 14 |
| Bacteria | Proteobacteria | Alphaproteobacteria | Rhizobiales | Phyllobacteriaceae | *Lentilitoribacter donghaensis* | 0 | 3 | 0 | 0 | 0 |
| Bacteria | Proteobacteria | Alphaproteobacteria | Rhizobiales | Phyllobacteriaceae | *Mesorhizobium australicum* | 0 | 0 | 3 | 0 | 0 |
| Bacteria | Proteobacteria | Alphaproteobacteria | Rhizobiales | Phyllobacteriaceae | *Mesorhizobium waimense* | 0 | 0 | 0 | 0 | 46 |
| Bacteria | Proteobacteria | Alphaproteobacteria | Rhizobiales | Phyllobacteriaceae | *Nitratireductor kimnyeongensis* | 0 | 3 | 0 | 0 | 0 |
| Bacteria | Proteobacteria | Alphaproteobacteria | Rhizobiales | Phyllobacteriaceae | *Oricola cellulosilytica* | 0 | 77 | 0 | 0 | 0 |
| Bacteria | Proteobacteria | Alphaproteobacteria | Rhizobiales | Phyllobacteriaceae | *Pseudaminobacter manganicus* | 0 | 0 | 0 | 16 | 8 |
| Bacteria | Proteobacteria | Alphaproteobacteria | Rhizobiales | Rhizobiaceae | *Pararhizobium haloflavum* | 0 | 0 | 0 | 3 | 0 |
| Bacteria | Proteobacteria | Alphaproteobacteria | Rhizobiales | Rhizobiaceae | *Sinorhizobium saheli* | 0 | 0 | 0 | 32 | 5 |
| Bacteria | Proteobacteria | Alphaproteobacteria | Rhizobiales | Rhodobiaceae | *Microbaculum marinum* | 0 | 0 | 63 | 0 | 1 |
| Bacteria | Proteobacteria | Alphaproteobacteria | Rhizobiales | Rhodobiaceae | *Tepidamorphus gemmatus* | 0 | 0 | 0 | 0 | 3 |
| Bacteria | Proteobacteria | Alphaproteobacteria | Rhizobiales | Salinarimonadaceae | *Salinarimonas ramus* | 6 | 0 | 0 | 11 | 47 |
| Bacteria | Proteobacteria | Alphaproteobacteria | Rhodobacterales | Hyphomonadaceae | *Fretibacter rubidus* | 0 | 1 | 996 | 1 | 0 |
| Bacteria | Proteobacteria | Alphaproteobacteria | Rhodobacterales | Hyphomonadaceae | *Henriciella litoralis* | 5 | 1 | 31 | 0 | 4 |
| Bacteria | Proteobacteria | Alphaproteobacteria | Rhodobacterales | Hyphomonadaceae | *Hyphobacterium vulgare* | 0 | 31 | 0 | 0 | 0 |
| Bacteria | Proteobacteria | Alphaproteobacteria | Rhodobacterales | Hyphomonadaceae | *Hyphomonas adhaerens* | 2 | 0 | 48 | 0 | 12 |
| Bacteria | Proteobacteria | Alphaproteobacteria | Rhodobacterales | Hyphomonadaceae | *Marinicauda algicola* | 0 | 0 | 0 | 0 | 6 |
| Bacteria | Proteobacteria | Alphaproteobacteria | Rhodobacterales | Rhodobacteraceae | *Aliishimia ponticola* | 0 | 0 | 0 | 0 | 3 |
| Bacteria | Proteobacteria | Alphaproteobacteria | Rhodobacterales | Rhodobacteraceae | *Donghicola eburneus* | 2077 | 1160 | 177 | 5191 | 136 |
| Bacteria | Proteobacteria | Alphaproteobacteria | Rhodobacterales | Rhodobacteraceae | *Labrenzia marina* | 0 | 0 | 0 | 1 | 4 |
| Bacteria | Proteobacteria | Alphaproteobacteria | Rhodobacterales | Rhodobacteraceae | *Marivita hallyeonensis* | 2 | 314 | 13 | 140 | 184 |
| Bacteria | Proteobacteria | Alphaproteobacteria | Rhodobacterales | Rhodobacteraceae | *Marivivens donghaensis* | 0 | 0 | 0 | 379 | 0 |
| Bacteria | Proteobacteria | Alphaproteobacteria | Rhodobacterales | Rhodobacteraceae | *Nereida ignava* | 0 | 927 | 0 | 0 | 1 |
| Bacteria | Proteobacteria | Alphaproteobacteria | Rhodobacterales | Rhodobacteraceae | *Oceanicella actignis* | 30 | 3 | 21 | 158 | 3 |
| Bacteria | Proteobacteria | Alphaproteobacteria | Rhodobacterales | Rhodobacteraceae | *Oceaniglobus indicus* | 40 | 0 | 332 | 102 | 151 |
| Bacteria | Proteobacteria | Alphaproteobacteria | Rhodobacterales | Rhodobacteraceae | *Octadecabacter ascidiaceicola* | 0 | 5 | 0 | 0 | 0 |
| Bacteria | Proteobacteria | Alphaproteobacteria | Rhodobacterales | Rhodobacteraceae | *Paracoccus homiensis* | 0 | 1 | 55 | 6 | 11 |
| Bacteria | Proteobacteria | Alphaproteobacteria | Rhodobacterales | Rhodobacteraceae | *Primorskyibacter aestuariivivens* | 0 | 335 | 0 | 0 | 0 |
| Bacteria | Proteobacteria | Alphaproteobacteria | Rhodobacterales | Rhodobacteraceae | *Pseudooceanicola lipolyticus* | 0 | 0 | 5 | 2 | 1 |
| Bacteria | Proteobacteria | Alphaproteobacteria | Rhodobacterales | Rhodobacteraceae | *Pseudoruegeria sabulilitoris* | 18 | 810 | 46 | 112 | 245 |
| Bacteria | Proteobacteria | Alphaproteobacteria | Rhodobacterales | Rhodobacteraceae | *Psychromarinibacter halotolerans* | 4 | 0 | 1 | 0 | 0 |
| Bacteria | Proteobacteria | Alphaproteobacteria | Rhodobacterales | Rhodobacteraceae | *Puniceibacterium confluentis* | 32 | 184 | 0 | 16 | 4 |
| Bacteria | Proteobacteria | Alphaproteobacteria | Rhodobacterales | Rhodobacteraceae | *Rhodovulum adriaticum* | 0 | 6 | 0 | 0 | 0 |
| Bacteria | Proteobacteria | Alphaproteobacteria | Rhodobacterales | Rhodobacteraceae | *Rhodovulum imhoffii* | 37 | 0 | 0 | 12 | 3 |
| Bacteria | Proteobacteria | Alphaproteobacteria | Rhodobacterales | Rhodobacteraceae | *Rhodovulum tesquicola* | 7 | 0 | 12 | 0 | 0 |
| Bacteria | Proteobacteria | Alphaproteobacteria | Rhodobacterales | Rhodobacteraceae | *Roseicyclus mahoneyensis* | 0 | 0 | 1 | 0 | 2 |
| Bacteria | Proteobacteria | Alphaproteobacteria | Rhodobacterales | Rhodobacteraceae | *Roseovarius algicolus* | 46 | 0 | 0 | 57 | 54 |
| Bacteria | Proteobacteria | Alphaproteobacteria | Rhodobacterales | Rhodobacteraceae | *Ruegeria marisrubri* | 1820 | 27 | 317 | 46 | 824 |
| Bacteria | Proteobacteria | Alphaproteobacteria | Rhodobacterales | Rhodobacteraceae | *Sedimentitalea todarodis* | 41 | 0 | 168 | 19 | 14 |
| Bacteria | Proteobacteria | Alphaproteobacteria | Rhodobacterales | Rhodobacteraceae | *Shimia biformata* | 469 | 1 | 1123 | 117 | 237 |
| Bacteria | Proteobacteria | Alphaproteobacteria | Rhodobacterales | Rhodobacteraceae | *Stappia taiwanensis* | 1 | 0 | 2 | 1 | 2 |
| Bacteria | Proteobacteria | Alphaproteobacteria | Rhodobacterales | Rhodobacteraceae | *Sulfitobacter noctilucae* | 0 | 245 | 0 | 0 | 0 |
| Bacteria | Proteobacteria | Alphaproteobacteria | Rhodobacterales | Rhodobacteraceae | *Sulfitobacter pontiacus* | 0 | 5 | 0 | 0 | 1 |
| Bacteria | Proteobacteria | Alphaproteobacteria | Rhodobacterales | Rhodobacteraceae | *Yoonia maricola* | 0 | 339 | 0 | 0 | 0 |
| Bacteria | Proteobacteria | Alphaproteobacteria | Rhodospirillales | Acetobacteraceae | *Gluconobacter cerevisiae* | 0 | 0 | 13 | 0 | 11 |
| Bacteria | Proteobacteria | Alphaproteobacteria | Rhodospirillales | Geminicoccaceae | *Arboricoccus pini* | 0 | 0 | 3 | 0 | 0 |
| Bacteria | Proteobacteria | Alphaproteobacteria | Rhodospirillales | Rhodospirillaceae | *Aestuariispira insulae* | 0 | 12 | 0 | 0 | 0 |
| Bacteria | Proteobacteria | Alphaproteobacteria | Rhodospirillales | Rhodospirillaceae | *Haematospirillum jordaniae* | 0 | 0 | 0 | 4 | 0 |
| Bacteria | Proteobacteria | Alphaproteobacteria | Rhodospirillales | Rhodospirillaceae | *Oceanibaculum pacificum* | 4 | 0 | 4 | 12 | 1 |
| Bacteria | Proteobacteria | Alphaproteobacteria | Rhodospirillales | Rhodospirillaceae | *Pelagibius litoralis* | 0 | 0 | 21 | 11 | 3 |
| Bacteria | Proteobacteria | Alphaproteobacteria | Rickettsiales | __ | *Lyticum flagellatum* | 0 | 6 | 0 | 0 | 0 |
| Bacteria | Proteobacteria | Alphaproteobacteria | Rickettsiales | Rickettsiaceae | *Rickettsia bellii* | 0 | 22 | 0 | 1 | 1 |
| Bacteria | Proteobacteria | Alphaproteobacteria | Rickettsiales | Rickettsiaceae | *Rickettsia conorii* | 0 | 9 | 0 | 0 | 0 |
| Bacteria | Proteobacteria | Alphaproteobacteria | Sphingomonadales | Erythrobacteraceae | *Altererythrobacter luteolus* | 0 | 63 | 0 | 0 | 0 |
| Bacteria | Proteobacteria | Alphaproteobacteria | Sphingomonadales | Erythrobacteraceae | *Erythrobacter gaetbuli* | 0 | 0 | 2 | 0 | 2 |
| Bacteria | Proteobacteria | Alphaproteobacteria | Sphingomonadales | Erythrobacteraceae | *Erythrobacter gangjinensis* | 0 | 0 | 26 | 0 | 2 |
| Bacteria | Proteobacteria | Alphaproteobacteria | Sphingomonadales | Erythrobacteraceae | *Porphyrobacter algicida* | 1 | 0 | 0 | 1 | 1 |
| Bacteria | Proteobacteria | Alphaproteobacteria | Sphingomonadales | Sphingomonadaceae | *Parasphingopyxis algicola* | 0 | 3 | 22 | 0 | 4 |
| Bacteria | Proteobacteria | Alphaproteobacteria | Sphingomonadales | Sphingomonadaceae | *Polymorphobacter fuscus* | 38 | 0 | 0 | 0 | 1 |
| Bacteria | Proteobacteria | Betaproteobacteria | Burkholderiales | Burkholderiaceae | *Limnobacter thiooxidans* | 0 | 0 | 0 | 7 | 4 |
| Bacteria | Proteobacteria | Betaproteobacteria | Burkholderiales | Burkholderiaceae | *Pandoraea oxalativorans* | 2 | 0 | 0 | 1 | 0 |
| Bacteria | Proteobacteria | Betaproteobacteria | Burkholderiales | Comamonadaceae | *Acidovorax monticola* | 0 | 18 | 0 | 0 | 0 |
| Bacteria | Proteobacteria | Betaproteobacteria | Nitrosomonadales | Methylophilaceae | *Methylotenera versatilis* | 0 | 23 | 0 | 0 | 0 |
| Bacteria | Proteobacteria | Betaproteobacteria | Nitrosomonadales | Nitrosomonadaceae | *Nitrosomonas aestuarii* | 2 | 0 | 20 | 10 | 14 |
| Bacteria | Proteobacteria | Deltaproteobacteria | Desulfobacterales | Desulfobacteraceae | *Desulfatibacillum alkenivorans* | 0 | 0 | 0 | 0 | 108 |
| Bacteria | Proteobacteria | Deltaproteobacteria | Desulfobacterales | Desulfobulbaceae | *Desulforhopalus singaporensis* | 0 | 10 | 0 | 0 | 0 |
| Bacteria | Proteobacteria | Deltaproteobacteria | Desulfuromonadales | Geobacteraceae | *Geoalkalibacter subterraneus* | 5298 | 12 | 0 | 0 | 54 |
| Bacteria | Proteobacteria | Deltaproteobacteria | Desulfuromonadales | Geobacteraceae | *Geobacter argillaceus* | 0 | 0 | 12 | 0 | 0 |
| Bacteria | Proteobacteria | Deltaproteobacteria | Myxococcales | __ | *Enhygromyxa salina* | 0 | 1 | 1 | 0 | 1 |
| Bacteria | Proteobacteria | Deltaproteobacteria | Myxococcales | Polyangiaceae | *Chondromyces lanuginosus* | 0 | 0 | 0 | 0 | 27 |
| Bacteria | Proteobacteria | Deltaproteobacteria | Myxococcales | Sandaracinaceae | *Sandaracinus amylolyticus* | 346 | 0 | 28 | 0 | 7 |
| Bacteria | Proteobacteria | Deltaproteobacteria | Syntrophobacterales | Syntrophaceae | *Desulfomonile limimaris* | 3 | 0 | 0 | 0 | 6 |
| Bacteria | Proteobacteria | Gammaproteobacteria | __ | __ | *Pseudohongiella nitratireducens* | 0 | 0 | 12 | 0 | 2 |
| Bacteria | Proteobacteria | Gammaproteobacteria | __ | __ | *Pseudohongiella spirulinae* | 0 | 3 | 0 | 0 | 1 |
| Bacteria | Proteobacteria | Gammaproteobacteria | __ | __ | *Sedimenticola thiotaurini* | 0 | 0 | 56 | 0 | 0 |
| Bacteria | Proteobacteria | Gammaproteobacteria | Alteromonadales | __ | *Motilimonas eburnea* | 51 | 2 | 126 | 28 | 2 |
| Bacteria | Proteobacteria | Gammaproteobacteria | Alteromonadales | Alteromonadaceae | *Alteromonas mediterranea* | 0 | 1097 | 0 | 1 | 1 |
| Bacteria | Proteobacteria | Gammaproteobacteria | Alteromonadales | Alteromonadaceae | *Alteromonas pelagimontana* | 0 | 3 | 0 | 0 | 0 |
| Bacteria | Proteobacteria | Gammaproteobacteria | Alteromonadales | Alteromonadaceae | *Glaciecola pallidula* | 0 | 31 | 0 | 0 | 0 |
| Bacteria | Proteobacteria | Gammaproteobacteria | Alteromonadales | Alteromonadaceae | *Mangrovitalea sediminis* | 0 | 0 | 0 | 1538 | 97 |
| Bacteria | Proteobacteria | Gammaproteobacteria | Alteromonadales | Alteromonadaceae | *Marinobacter profundi* | 0 | 0 | 3 | 3 | 0 |
| Bacteria | Proteobacteria | Gammaproteobacteria | Alteromonadales | Colwelliaceae | *Thalassotalea fusca* | 0 | 25 | 0 | 2 | 2 |
| Bacteria | Proteobacteria | Gammaproteobacteria | Alteromonadales | Idiomarinaceae | *Idiomarina piscisalsi* | 0 | 0 | 0 | 0 | 35 |
| Bacteria | Proteobacteria | Gammaproteobacteria | Alteromonadales | Pseudoalteromonadaceae | *Pseudoalteromonas gelatinilytica* | 27 | 408 | 132 | 36 | 87 |
| Bacteria | Proteobacteria | Gammaproteobacteria | Alteromonadales | Pseudoalteromonadaceae | *Pseudoalteromonas piratica* | 8 | 32 | 32 | 35 | 11 |
| Bacteria | Proteobacteria | Gammaproteobacteria | Alteromonadales | Psychromonadaceae | *Psychromonas profunda* | 15 | 0 | 0 | 0 | 0 |
| Bacteria | Proteobacteria | Gammaproteobacteria | Cellvibrionales | Cellvibrionaceae | *Agarilytica rhodophyticola* | 0 | 0 | 6 | 0 | 0 |
| Bacteria | Proteobacteria | Gammaproteobacteria | Cellvibrionales | Cellvibrionaceae | *Gilvimarinus chinensis* | 0 | 0 | 1 | 7 | 0 |
| Bacteria | Proteobacteria | Gammaproteobacteria | Cellvibrionales | Halieaceae | *Congregibacter litoralis* | 0 | 7 | 0 | 0 | 0 |
| Bacteria | Proteobacteria | Gammaproteobacteria | Cellvibrionales | Halieaceae | *Halioglobus lutimaris* | 0 | 0 | 0 | 2 | 1 |
| Bacteria | Proteobacteria | Gammaproteobacteria | Cellvibrionales | Halieaceae | *Luminiphilus syltensis* | 0 | 6 | 0 | 0 | 0 |
| Bacteria | Proteobacteria | Gammaproteobacteria | Cellvibrionales | Halieaceae | *Parahaliea aestuarii* | 0 | 0 | 0 | 0 | 3 |
| Bacteria | Proteobacteria | Gammaproteobacteria | Cellvibrionales | Porticoccaceae | *Porticoccus hydrocarbonoclasticus* | 0 | 13 | 0 | 0 | 0 |
| Bacteria | Proteobacteria | Gammaproteobacteria | Chromatiales | __ | *Thiohalobacter thiocyanaticus* | 0 | 0 | 144 | 0 | 9 |
| Bacteria | Proteobacteria | Gammaproteobacteria | Chromatiales | Ectothiorhodospiraceae | *Natronocella acetinitrilica* | 3 | 0 | 0 | 0 | 0 |
| Bacteria | Proteobacteria | Gammaproteobacteria | Chromatiales | Granulosicoccaceae | *Sulfuriflexus mobilis* | 0 | 0 | 54 | 0 | 0 |
| Bacteria | Proteobacteria | Gammaproteobacteria | Chromatiales | Thioprofundaceae | *Thioprofundum hispidum* | 0 | 0 | 63 | 0 | 0 |
| Bacteria | Proteobacteria | Gammaproteobacteria | Chromatiales | Thioprofundaceae | *Thioprofundum lithotrophicum* | 5 | 0 | 69 | 12 | 62 |
| Bacteria | Proteobacteria | Gammaproteobacteria | Chromatiales | Wenzhouxiangellaceae | *Wenzhouxiangella marina* | 1 | 0 | 1 | 0 | 1 |
| Bacteria | Proteobacteria | Gammaproteobacteria | Chromatiales | Wenzhouxiangellaceae | *Wenzhouxiangella salilacus* | 76 | 0 | 2 | 105 | 263 |
| Bacteria | Proteobacteria | Gammaproteobacteria | Chromatiales | Woeseiaceae | *Woeseia oceani* | 139 | 0 | 897 | 157 | 534 |
| Bacteria | Proteobacteria | Gammaproteobacteria | Legionellales | Legionellaceae | *Legionella pneumophila* | 0 | 0 | 4 | 3 | 0 |
| Bacteria | Proteobacteria | Gammaproteobacteria | Legionellales | Legionellaceae | *Legionella thermalis* | 0 | 0 | 37 | 0 | 0 |
| Bacteria | Proteobacteria | Gammaproteobacteria | Oceanospirillales | Alcanivoracaceae | *Marinicella pacifica* | 1099 | 0 | 1 | 264 | 166 |
| Bacteria | Proteobacteria | Gammaproteobacteria | Oceanospirillales | Hahellaceae | *Hahella chejuensis* | 26 | 0 | 0 | 0 | 0 |
| Bacteria | Proteobacteria | Gammaproteobacteria | Oceanospirillales | Kangiellaceae | *Kangiella chungangensis* | 76 | 4 | 0 | 0 | 56 |
| Bacteria | Proteobacteria | Gammaproteobacteria | Oceanospirillales | Kangiellaceae | *Kangiella taiwanensis* | 0 | 0 | 0 | 14 | 90 |
| Bacteria | Proteobacteria | Gammaproteobacteria | Oceanospirillales | Litoricolaceae | *Litoricola marina* | 0 | 12 | 0 | 0 | 0 |
| Bacteria | Proteobacteria | Gammaproteobacteria | Oceanospirillales | Oceanospirillaceae | *Bacterioplanoides pacificum* | 0 | 10 | 0 | 0 | 0 |
| Bacteria | Proteobacteria | Gammaproteobacteria | Oceanospirillales | Saccharospirillaceae | *Reinekea blandensis* | 3 | 0 | 0 | 0 | 0 |
| Bacteria | Proteobacteria | Gammaproteobacteria | Oceanospirillales | Saccharospirillaceae | *Saccharospirillum impatiens* | 1 | 0 | 13 | 0 | 1 |
| Bacteria | Proteobacteria | Gammaproteobacteria | Pseudomonadales | Pseudomonadaceae | *Permianibacter aggregans* | 0 | 0 | 0 | 3 | 0 |
| Bacteria | Proteobacteria | Gammaproteobacteria | Pseudomonadales | Pseudomonadaceae | *Pseudomonas cedrina* | 0 | 0 | 0 | 478 | 41 |
| Bacteria | Proteobacteria | Gammaproteobacteria | Vibrionales | Vibrionaceae | *Vibrio alginolyticus* | 2 | 207 | 66 | 38 | 71 |
| Bacteria | Proteobacteria | Gammaproteobacteria | Vibrionales | Vibrionaceae | *Vibrio tritonius* | 4 | 0 | 5 | 10 | 31 |
| Bacteria | Proteobacteria | Gammaproteobacteria | Xanthomonadales | Xanthomonadaceae | *Lysobacter spongiae* | 0 | 0 | 28 | 0 | 0 |
| Bacteria | Proteobacteria | Oligoflexia | Bacteriovoracales | Bacteriovoracaceae | *Peredibacter starrii* | 0 | 0 | 0 | 2 | 1 |
| Bacteria | Proteobacteria | Oligoflexia | Bacteriovoracales | Halobacteriovoraceae | *Halobacteriovorax marinus* | 0 | 28 | 0 | 71 | 0 |
| Bacteria | Proteobacteria | Oligoflexia | Bdellovibrionales | Bdellovibrionaceae | *Bdellovibrio exovorus* | 0 | 12 | 0 | 11 | 0 |
| Bacteria | Proteobacteria | Oligoflexia | Oligoflexales | Oligoflexaceae | *Oligoflexus tunisiensis* | 0 | 0 | 0 | 0 | 67 |
| Bacteria | Proteobacteria | Oligoflexia | Silvanigrellales | Silvanigrellaceae | *Silvanigrella aquatica* | 0 | 0 | 0 | 7 | 25 |
| Bacteria | Tenericutes | Mollicutes | Acholeplasmatales | Acholeplasmataceae | *Acholeplasma parvum* | 0 | 7 | 0 | 0 | 0 |
| Bacteria | Verrucomicrobia | Verrucomicrobiae | Verrucomicrobiales | Rubritaleaceae | *Rubritalea halochordaticola* | 2 | 0 | 0 | 11 | 0 |
| Bacteria | Verrucomicrobia | Verrucomicrobiae | Verrucomicrobiales | Verrucomicrobiaceae | *Haloferula helveola* | 0 | 2 | 3 | 50 | 141 |
| Bacteria | Verrucomicrobia | Verrucomicrobiae | Verrucomicrobiales | Verrucomicrobiaceae | *Luteolibacter algae* | 0 | 137 | 392 | 7 | 2 |
| Bacteria | Verrucomicrobia | Verrucomicrobiae | Verrucomicrobiales | Verrucomicrobiaceae | *Luteolibacter flavescens* | 0 | 0 | 0 | 2 | 1 |
| Bacteria | Verrucomicrobia | Verrucomicrobiae | Verrucomicrobiales | Verrucomicrobiaceae | *Roseibacillus ishigakijimensis* | 0 | 0 | 0 | 0 | 3 |
| Bacteria | Verrucomicrobia | Verrucomicrobiae | Verrucomicrobiales | Verrucomicrobiaceae | *Roseibacillus persicicus* | 0 | 0 | 0 | 81 | 254 |
| Bacteria | Verrucomicrobia | Verrucomicrobiae | Verrucomicrobiales | Verrucomicrobiaceae | *Roseibacillus ponti* | 0 | 20 | 0 | 4 | 0 |
| Total frequency | | | | | | 24,636 | 41,533 | 25,421 | 22,918 | 21,321 |

The microbial species detected in at least one of the five samples are shown. Unclassified taxonomic names (phylum, class, order, family, and species) are shown by __.

**TABLE S3** Illumina MiSeq sequencing results for the operational taxonomic units (OTUs) and statistical diversity analysis in the five tanks from the studied *Litopenaeus vannamei* shrimp farms.

|  |  | Tank A | | Tank B | | Tank C | | Tank D | | Tank E | |
| --- | --- | --- | --- | --- | --- | --- | --- | --- | --- | --- | --- |
|  |  | Eu^a^ | Pro^b^ | Eu^a^ | Pro^b^ | Eu^a^ | Pro^b^ | Eu^a^ | Pro^b^ | Eu^a^ | Pro^b^ |
| Sequencing results | Total reads | 98041 | 24708 | 90416 | 41788 | 102353 | 26926 | 105366 | 23873 | 120981 | 22060 |
|  | Validated reads | 23414 | 24636 | 70926 | 41533 | 62771 | 25421 | 62095 | 22918 | 24406 | 21321 |
|  | Mean read length (bp) | 402.90 | 438.93 | 414.55 | 450.89 | 410.56 | 438.33 | 416.43 | 439.43 | 407.07 | 438.63 |
|  | Maximum read length (bp) | 419 | 461 | 419 | 461 | 419 | 461 | 419 | 461 | 419 | 461 |
|  | Number of OTUs^c^ | 48 | 141 | 54 | 115 | 152 | 188 | 44 | 183 | 87 | 228 |
| Diversity indicators | Chao1^d^ | 48.00 | 149.75 | 54.00 | 137.75 | 154.50 | 207.00 | 44.00 | 206.63 | 93.00 | 276.24 |
|  | Shannon^e^ | 2.37 | 4.35 | 2.00 | 3.11 | 2.83 | 5.03 | 1.36 | 4.52 | 1.55 | 4.56 |
|  | Inverse simpson^f^ | 0.73 | 0.90 | 0.57 | 0.68 | 0.68 | 0.92 | 0.53 | 0.91 | 0.41 | 0.88 |
|  | Goods Coverage^g^ | 1.00 | 1.00 | 1.00 | 1.00 | 1.00 | 1.00 | 1.00 | 1.00 | 1.00 | 1.00 |

^a^Eu: Eukaryotic microbial community.

^b^Pro: Prokaryotic microbial community.

^c^OTUs: Operational taxonomic units.

^d^Chao1: Species richness estimation.

^e^Shannon: Shannon diversity index (>0, higher is more diverse).

^f^Inverse Simpson: Inverted simpson diversity index (0–1, 0 = most simple).

^g^Goods Coverage: 1 – (number of singleton OTUs/number of sequences), 1 = 100% coverage.
